# Geographical Distribution of Carnivore Hosts and Genotypes of Canine Distemper Virus (CDV) Worldwide: A Scoping Review and Spatial Meta-Analysis

**DOI:** 10.1155/tbed/6632068

**Published:** 2025-03-05

**Authors:** A. Wipf, P. Perez-Cutillas, N. Ortega, A. Huertas-López, C. Martínez-Carrasco, M. G. Candela

**Affiliations:** ^1^Animal Health Department, Faculty of Veterinary, University of Murcia, Murcia, Spain; ^2^Geography Department, Faculty of Humanities, University of Murcia, Murcia, Spain; ^3^SALUVET, Animal Health Department, Faculty of Veterinary, Complutense University of Madrid, Madrid, Spain

**Keywords:** Carnivora, CDV, genotype, morbillivirus, multihost, spatial meta-analysis, spillover

## Abstract

**Background:** Emerging viral diseases are spreading to new geographic locations, influenced by human activities and climate change. *Morbillivirus canis* (also known as canine distemper virus, CDV), the etiological agent of CD, is distributed worldwide and shared between wild and domestic animals.

**Methods:** A systematic review using MeSH terms was carried out from 1985 to 2024, focusing the search on studies (PubMed and WOS) that would detect CDV and sequence it in a known genotype in Carnivora hosts. Articles were reviewed by four researchers, and after quality assessment, we selected 160 published papers for data extraction, analysis, and spatial meta-analysis. Considering species studied, geographical location, and classified genotypes we identify 457 different individual studies (records) from which 332 records CDV was sequenced into a classifiable 17 main genotypes. Spatial meta-analysis was performed using QGIS, revealing distributions of animals in which a CDV lineage has been isolated; geographical lineages overlapping on different hosts have been measured as a density function.

**Results:** CDV host species belonged to the suborder Caniformia (93.7%) into families such as Canidae (75.2%), Mustelidae (9.7%), and Procyonidae (7.6%). Suborder Feliformia (6.1%) showed wild Felidae (5.1%) as the most represented family. Samples used were brain (13.74%), lung (12.4%), blood (10.8%), and nasal-eye discharges (8.9%; 8.1%). Reverse transcription-PCR (RT-PCR) (60.34%) and real-time-quantitative PCR (RT-qPCR) (26.57%) detecting *H* gene (62%) were most used to detect viral ARN. On genotypes, Europe/South America-1 (27.4%), Europe-3/Artic (15.5%), Asia-1 (14.5%), America-1 (11.2%), Europe-2/European Wildlife and Africa (Africa-1 and Africa-2) (7.6%) were the most represented worldwide, being America-1 and Europe/South America-1 the most widely distributed around the world.

**Conclusions:** The analysis showed the wide multihost capacity and diversity of CDV, with dog (*Canis lupus familiaris*) as the most frequent (40%) and red fox (*Vulpes vulpes*) (30.2%) as the main wild host. Most of the detected lineages can be detected in several wild host families, in addition to the dog, suggesting constant spillover phenomena in shared habitats at the domestic–wild interface. The most cosmopolitan lineages mirror the distribution routes of their hosts, showing that it is difficult to establish a CDV-fixed picture in an interconnected world.

## 1. Introduction

*Morbillivirus canis*, also known as canine distemper virus (CDV), is a morbillivirus belonging to the *Paramyxoviridae* family that causes a highly contagious, acute, and fatal disease, CD [1]. This RNA virus measures 150–250 nm and has a lipoprotein envelope [2]. CDV is a multihost pathogen with a worldwide distribution, affecting mainly a wide range of wild and domestic species of the Carnivora order. The dog (*Canis lupus familiaris*) is considered the main reservoir host for CDV, although many wild carnivore species can act as reservoirs for CDV and also suffer from CD [2]. In fact, spillover from dog reservoirs to wildlife species has led to high mortality outbreaks that represent a major conservation threat [3]. CDV has been described in families of wild carnivores such as Canidae, Mustelidae, Procyonidae, Mephitidae, Phocidae, Ursidae, Felidae, Hyaenidae, and Viverridae [2, 4–6]. In addition, it has been described in aquatic mammals [7], nonhuman primates belonging to Cercopithecidae and other families [8–10], other mammals belonging to Myrmecophagidae [11], Hystricidae and Sciuridae families [12], Artiodactyla [13], and Proboscidea orders [14, 15].

CDV can produce a fatal multisystemic disease affecting the immune, respiratory, gastrointestinal, and nervous systems where common outcomes of infection include lymphoid depletion, hyperkeratosis, interstitial pneumonia (often complicated by opportunistic bacterial infections), encephalopathy, which may result in death [16–20]. Virions are highly contagious, transmitted through aerosolized nasal, oral, and ocular fluid, and its major route of entry for infection is through the respiratory system [21, 22]. CDV is unstable in the environment and relies on a supply of new hosts to persist among animal populations [23].

The CDV genome encodes eight viral proteins, two of which are nonstructural (C and V) and six are structural: hemagglutinin (H), fusion (F), matrix (M), phosphoprotein (P), large polymerase (L), and nucleocapsid (N) [24]. H and F glycoproteins are responsible for virus attachment to and fusion with the host cells [25]. These two glycoproteins are more variable than other CDV proteins and possess the major antigenic determinants that induce protective immune responses against CDV [25, 26]. These features make H and F proteins suitable for genetic lineage identification, phylogenetic analysis, and useful markers for evolutionary studies [27, 28]. In fact, sequence analysis of the *H* and *F* genes (of which the Fsp-coding region shows the highest variability) has been widely studied and employed to characterize CDV field strains worldwide, revealing a geographical pattern of genetic diversity [25, 29–32].

Till 2014, nine genetic lineages were reported based on H protein sequence identity (more than 95%) [33]. Since then, however, the number of CDV lineages accepted in the scientific literature has shown a steady increase. Currently, most authors work with 17 genotypes to classify CDV [10, 14]. However, some authors raise this number to 21 lineages [34]. Genetic lineages are a constantly revisited topic, as it is common for researchers to propose new lineages based on circulating strains from certain areas of the world [35].

The main genotypes into which most of the CDV strains detected worldwide are classified include America-1 (including Western vaccines), America-2 to America-4, Europe 1 (which includes genotypes reported from 2006 [36] to 2023 years [37]), Europe/South America-1, South America-2, South America-3, Europe-2/European Wildlife, Europe-3/Arctic, Asia-1 to Asia-6, Africa-1, and Africa-2. The number of genetic lineages has reached 17, namely America 1 to America 5 (America 1 includes commercially available vaccines), Asia 1 to Asia 5, Europe/South America 1, South America 2 and 3, European Wildlife, Arctic, Africa 1 and Africa 2.

Dogs are known as the main domestic reservoir of *Morbillivirus canis*, although as a multihost pathogen, it can also affect other mammal carnivores, where epidemic outbreaks with virus circulation among several species are often described, highlighting the strong adaptation of CDV strains to wild susceptible hosts [15, 38–44]. So far, most CVD studies in wildlife have focused on indirect detection of the virus through serological tests, but very few have addressed whether there is a relationship between the host species and virus lineage [14, 15, 30, 45–47]. Molecular epidemiology, based on partial or whole genome sequencing (WGS) of CDV genomes, has been very useful in tracing the origin of CDV strains and now allows us to trace the global dynamics of circulation of different strains in different susceptible host species.

Recently, an increasing number of studies on CDV and other viruses that evaluate the spatio-temporal dynamics of viruses have been carried out [48, 49]. These types of studies are useful to increase the knowledge of the spread of the viruses worldwide and the potential mechanisms explaining that spread. However, from the authors' knowledge, before 2015 [33], the spatial distribution of the CDV strains has only been assessed at a local scale, normally focused on one host species, or with paleopathological criteria to elucidate aspects of the origin of the CDV [50].

The present study aimed to conduct a scoping review and spatial meta-analysis at the global level, focusing on wild and domestic species of the order Carnivora, analyzing the molecular detection of *Morbillivirus canis* and its phylogenetic classification in the main CDV lineages in order to answer two questions: (i) whether the detection of different CDV genetic lineages exhibits any pattern that is related to the wild or domestic host in which it is detected, and (ii) whether the detection of different CDV genetic lineages exhibits any pattern that is related to the geographical location in which that strain has been detected.

## 2. Material and Methods

These scoping reviews and spatial meta-analyses were conducted according to the Preferred Reporting Items for Systematic Reviews and Meta-Analyses Extension for Scoping Reviews (PRISMA-Scr) statement [51]. The PRISMA-Scr checklist is shown in Supporting Information 1. The systematic search, the quality assessment, and the data extraction were performed by four independent researchers. Data were cross-checked and disagreements were solved by a senior researcher. The review protocol was registered at the OSF platform (date 02/05/2025; access: osf.io/x7ewh).

### 2.1. Systematic Search

**Figure 1:**
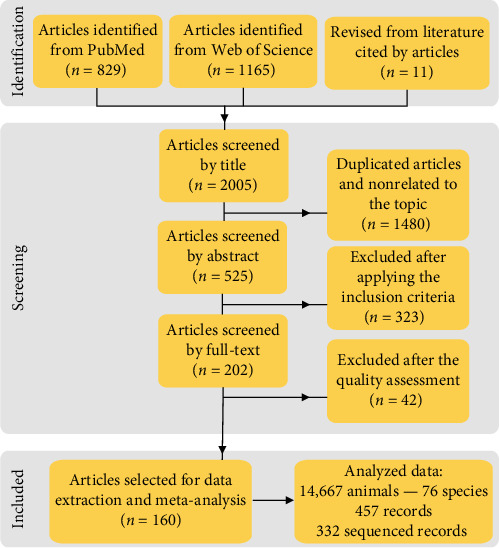
Flow diagram for the selection of articles.

In brief, a systematic search on the scientific databases Web of Science and PubMed was performed from 1985 to December 2024 (search date 12/21/2024). The following MeSH terms were employed: “(Canine distemper virus OR Morbillivirus canis OR Canine Morbillivirus) AND (Carnivore) AND (PCR OR Sequencing OR Phylogeny OR Phylogenetic OR Phylogenomic OR Genetic OR Characterization OR Genetic lineage OR Hemagglutinin).” The data were double-checked for all articles included in the qualitative synthesis. Initially, 1994 articles were found in the scientific databases, and the revision of their reference lists resulted in the inclusion of other 11 articles (Figure 1). So, a total of 2005 articles were identified. Articles were reviewed at successive levels by title, abstract, and full text. After removing duplicated articles using a single EndNote file (EndNote Web 21, Clarivate Analytics, Philadelphia, PA, USA), the following inclusion criteria were applied:

1. Studies performed on domestic or wild carnivores.
2. Studies molecularly detecting CDV and sequencing the detected strain.
3. Articles written in Spanish, Portuguese, and English.

The quality of the 202 studies that met the inclusion criteria was assessed. The following parameters were considered for the quality assessment:

• PCR procedure (number of cycles, denaturation temperature, primer annealing temperature, and primer extension temperature).
• Type of PCR (real-time-quantitative PCR [RT-qPCR], reverse transcription-PCR [RT-PCR], nested RT-PCR, heminested RT-PCR).
• Information detailed about target gen used for CDV detection and sequencing.
• Information detailed about primers used.
• Use of negative extraction controls and positive controls to monitor PCR inhibitors.
• Product detection method.

The lack of information regarding these parameters that could limit the reproducibility of the results was considered a “high risk of bias” and, therefore, excluded from the subsequent data extraction and spatial meta-analysis. A total of 160 articles were finally selected for data extraction and meta-analysis (Figure 1). The data were independently extracted by four researchers using a standardized Excel spreadsheet (Microsoft Excel for Microsoft 365 MSO, Version 2307 built 16.0.16626.20170).

To analyze the data from the articles that met the criteria established for inclusion in this scoping review, we worked with different layers of information, including (Figure 1):

• Articles: Total number of articles reviewed (Supporting Information 2).
• Animal: Total number of animals and species analyzed in the included articles.
• Records: Categorization that classifies the different individual studies analyzed in the articles reviewed. To work with and identify these individual studies, we considered the following criteria: (i) all the different species analyzed in each revised article, (ii) all the different genotypes detected by species in each article, (iii) all the different geographical areas from which the species originate and the different genotypes analyzed in each study.
• Sequenced records: Individual studies in which the CDV was detected and sequenced were classified into a main genotype.

A total of 17 variables were analyzed related to (i) host species, (ii) analytical methods employed, (iii) CDV genotypes considered, and (iv) date and geographical data (Table 1). Host species were classified into suborders Caniformia (Canidae, Mephitidae, Mustelidae, Procyonidae, Phocidae, and Ursidae families) and Feliformia (Felidae, Herpestidae, Hyaenidae, and Viverridae families) (Table 2).

CDV lineages were grouped following Panzera et al. [33] and Duque-Valencia et al. [14], in alphabetical order (Table 1), America-1 (including Western vaccine, Rockborn like and North America-1 genotypes); America-2; America-3 (including North America-3 genotypes); America-4 (including South America/North America-4); Europe/South America-1 (which includes genotypes reported as Europe-1 (from 2006 [36] to 2023 years [37], and other genotypes reported as Europe/South America-1: from 2006 [31] to 2023 [56]), South America-2 (including South America 2 Argentina and other strains from Argentina); South America-3 (including Colombia strains); Europe 2/European Wildlife (including Europe-2 genotypes); Europe-3/Arctic (including Arctic-like genotypes); Asia-1; Asia-2; Asia-3; Asia-4; Asia-5; Asia-6; Africa-1 (which includes Africa-1/Southern Africa genotypes); Africa-2 (including Africa-2/Eastern Africa genotypes).

### 2.2. Spatial Analysis

To determine spatial interactions in the CDV data, several tests were carried out using the Geographic Information System QGIS v.3.22 [57]. First, a geographical layout was developed to locate the host samples as well as the genotypes extracted in the review. These data were evaluated by species suborder (Caniformia/Feliformia) and grouped into domestic and wild hosts. In addition, to reveal the spatial distribution of genotypes, density estimation was carried out using Ripley's kernel function, applied to sequenced and classified CDV-positive animals [58, 59].

## 3. Results

### 3.1. Systematic Search

After the systematic search of the different scientific databases and the exclusion of 1480 duplicated articles and 323 articles that did not meet the inclusion criteria, 202 articles were preliminary selected (Figure 1). Moreover, 20.79% (42/202) of the articles with a high risk of bias according to the quality assessment were excluded. Therefore, a total of 160 articles from 1985 to December 2024 were finally selected (Figure 1, Supporting Information 2), from which 457 CDV records in the database were obtained for the subsequent data extraction, and host and spatial meta-analysis.

### 3.2. Host and Sampling Diversity

A total of 160 articles published between 1995 and 2024 were analyzed, with a total of 14,667 animals analyzed. Information on 457 individual records (different species, CDV genotypes, geographical locations worldwide) was extracted from these articles. Of these records, 398 (87.1%) had positive molecular detection of CDV, and of these, 332 (83.4%) were able to classify the sequenced RNA into one of the major genotypes, and 66 (16.6%) sequenced records could not be classified into one of the main genotypes. Related to species studied in the reviewed articles in which there was no molecular detection of CDV (59 (12.9%), Table 2), 8/28 species belong to the suborder Feliformia (*Acinonyx jubatus*, *Felis silvestris*, *Puma concolor*, *Leptailurus serval*, *Herpestes ichneumon*, *Mungo mungo*, *Rhynchogale melleri*, *Proteles cristatus*), while only 3/48 species belong to the suborder Caniformia (*Lycalopex gymnocercus*, *Mustela nivalis*, *Mustela erminea*).

**Figure 2:**
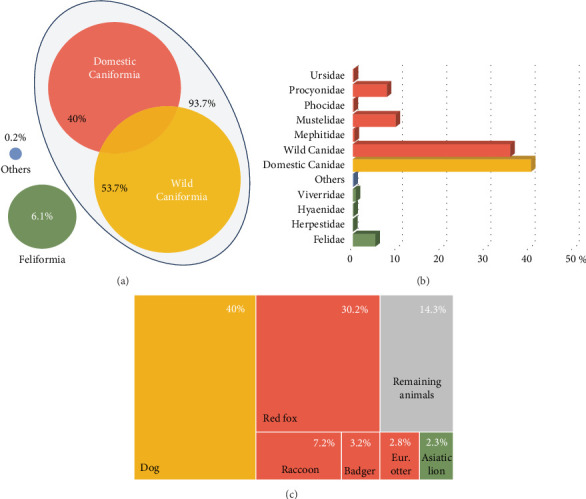
Percentage of animals analyzed (*n* = 14,467) in the 160 scientific articles reviewed. (A) Values of the Caniformia and Feliformia analyzed suborders. (B) Values categorized by carnivore family. (C) Values of the major occurring species.

The articles reviewed analyzed the CDV of two different mammal carnivore suborders, Caniformia and Feliformia; these suborders, families, and species of the carnivores studied are represented in Figure 2. Regarding the 14,667 analyzed animals, most of the records belonged to the Caniformia suborder (93.75%), while only 6.07% corresponded to Feliformia (Table 2; Figure 2A). A small percentage of animals could not be categorized (others, 0.17%) (Table 2; Figure 2A). Belonging to Caniformia, animals were categorized as domestic, with only dogs (*C. lupus familiaris*) as species (42.67%) or wild (57.33%) (Table 2; Figure 2A). The most represented families belonging to Caniformia were Canidae (75.25%), followed by Mustelidae (9.71%). Regarding species, domestic carnivores (40%) were the most analyzed hosts, followed by red fox (*Vulpes vulpes*) (30.23%) and raccoon (*Procyon lotor*) (7.23%). With lower percentages but standing out from the rest of the species of the suborder Caniformia are gray wolf (*C. lupus*) (1.52%) and raccoon dog (*Nyctereutes procyonoides*) (1.04%) in the family Canidae and badger (*Meles meles*) (1.55%), otter (*Lutra lutra*) (2.82%), and European polecat (*Mustela putorius*) (1.53%) in the family Mustelidae (Table 2; Figure 2B,C).

All animals classified as Feliformia were wild species because no domestic cat has been analyzed in the reviewed articles. The most analyzed families were Felidae (5.13%) and Viverridae (0.723%), with an occasional occurrence of Herpestidae and Hyaenidae. Focusing on the particular species, the most frequently analyzed were the Asiatic lion (*Panthera leo persica*) (2.24%) and the Iberian lynx (*Lynx pardinus*) (1.12%) (Table 2; Figure 2B,C).

**Figure 3:**
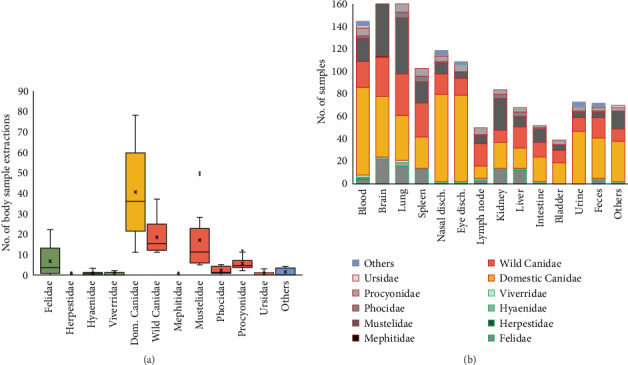
(A) Distribution of body samples number by carnivore family studied in samples analyzed in 457 analyzed records. (B) Body samples analyzed by the carnivore family; the red boxes in the legend indicate the families within the Caniformia suborder, and the green boxes indicate the families within the Feliformia suborder. *Note:* Several body samples could be used in a single record.

Regarding the type of sample obtained for CDV detection by PCR, the largest amount of sample extractions was provided by dogs (Figure 3A), and this species also showed the widest sampling diversity (Figure 3B). Other studies also sampled wild Canidae and Mustelidae families, but they represent approximately half of those dogs (Figure 3A). On the other hand, the Felidae family, although in a smaller proportion than Canidae (Figure 3A), shows a wide diversity of samples used (Figure 3B). In the present review, the most used samples in 457 analyzed records were brain (13.74%), lung (12.4%), and blood (10.8%), followed by nasal and eye discharges (8.9% and 8.1%, respectively), and by spleen (7.7%), kidney (6.3%) and urine (5.4%) (Figure 3B).

### 3.3. Analytical Methods Employed

**Figure 4:**
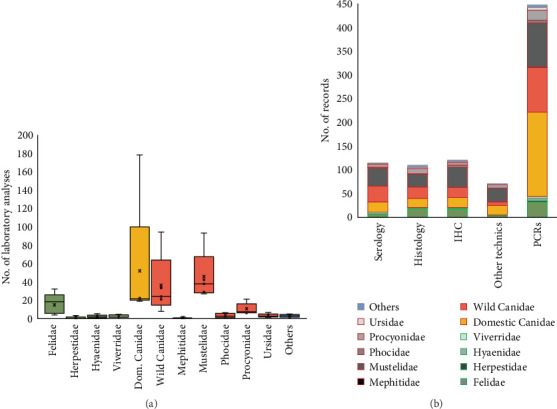
(A) Distribution of laboratory analyses done by carnivore family studied in 457 analyzed records. (B) Analytical methods employed for CDV detection conducted among carnivore families; the red boxes in the legend indicate the families within the Caniformia suborder, and the green boxes indicate the families within the Feliformia suborder. *Note:* Several analytical methods could be used in a single record. CDV, canine distemper virus.

Dogs were the host species subjected to the most diverse laboratory diagnostic techniques used, followed by Mustelidae and wild Canidae families (Figure 4A). In addition to molecular PCR techniques, different complementary techniques for indirect/direct detection of CDV were employed in some studies, including immunohistochemical (ICH) techniques (14.0%), serology (13.3%), or histology (12.7%) (Figure 4B). These techniques were complementary to PCR (for direct detection of CDV), which were used in all the articles (457 records). All techniques were used in both wild and domestic carnivores (Figure 4B).

**Figure 5:**
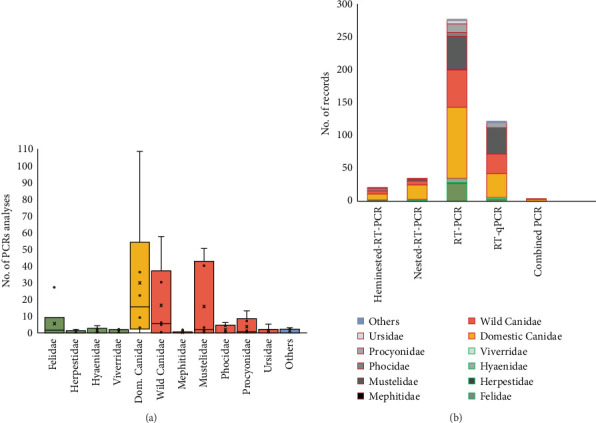
(A) Distribution of PCR analyses done by carnivore family studied in 457 analyzed records. (B) Type of PCR techniques done by the carnivore family studied; the red boxes in the legend indicate the families within the Caniformia suborder, and the green boxes indicate the families within the Feliformia suborder. *Note:* Several PCR techniques could be used in a single record.

Among types of PCR techniques, dogs showed by far the wider variability of PCR techniques among the species studied, followed by Mustelidae, wild Canidae and Procyonidae families (Figure 5A). Frequencies of the different types of PCR techniques used for CDV detection in the reviewed 457 records are shown in Figure 5B, with the most common being RT-PCR (60.34%) and RT-qPCR (26.57%). Some studies also applied nested RT-PCR (7.6%), hemi-nested RT PCR (4.5%), and combined PCR (0.8%).

### 3.4. Genotype Analysis

**Figure 6:**
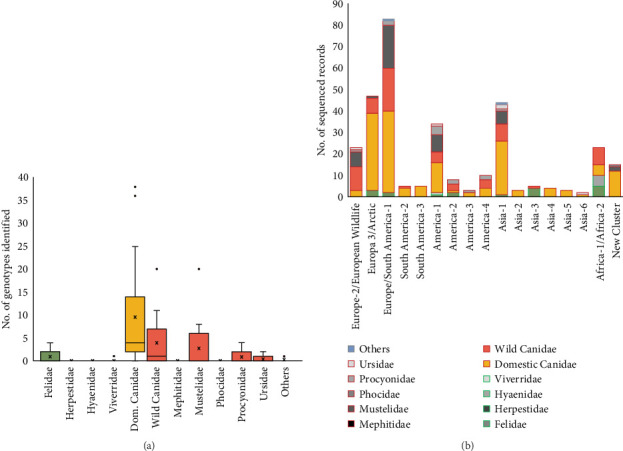
(A) Distribution of the genotype number identified by carnivore family studied in 332 sequenced records (records in which the CDV was sequenced). (B) Main genotypes identified by carnivore family studied in 332 sequenced records; the red boxes in the legend indicate the families within the Caniformia suborder, and the green boxes indicate the families within the Feliformia suborder. CDV, canine distemper virus.

We analyzed which CDV lineages were sequenced and in which hosts they were detected. Of the 457 records reviewed, CDV was detected in 398 (87.1%), of which 332 (83.4%) records sequenced the genetic material and classified into one of the 17 major lineages defined. The genotypes Europe/South America-1 (27.4%), Europe-3/Artic (15.5%), Asia-1 (14.5%), America-1 (11.2%), Europe-2/European Wildlife and Africa (Africa-1 and Africa-2) (7.6%) were the most represented genotypes worldwide (Figure 6B, Supporting Information 3).

**Figure 7:**
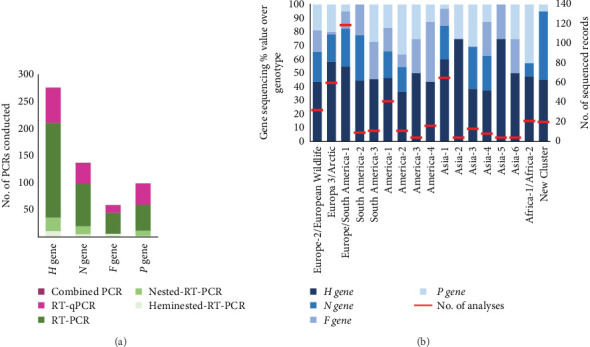
(A) Different CDV genes detected, and PCRs techniques used, in 457 analyzed records. (B) The left axis shows percentage values of genes used in 332 records in which the CDV was sequenced in a main genotype; the red line in the right axis represents the number of the main genotypes reported. CDV, canine distemper virus.

The reviewed records sequenced different CDV genes, with the *H* gene being by far the most widely used (51.8%). This is followed by the *N* gene (22.4%), while the *P* and *F* genes showed a similar frequency of use (13.5% and 12.2%, respectively) for CDV sequencing, being the genes of choice when using when hemi-nested RT-PCR was used for detection (Figure 7A). The majority of PCR techniques that detected the *H* gene were both RT-PCR and RT-qPCR, which were also used for detecting *N* and *P* genes. Regarding the gene that each of the 457 records used to sequence every specific CDV lineage (Figure 7B), it is shown that the *H* gene was the most commonly used for sequencing the majority of the CDV lineages; it was the predominant gene selected to detect the Asia-2, Asia-5, Asia-1, Europe-3/Arctic, Europe/South America-1, America-3, and Asia-6 (75%, 75%, 60%, 58.3%, 54.6%, 50%, and 50%, respectively). In the rest of the genotypes, the % use of the *H* gene is less than 50%. To detect the America-1 (genotype, including vaccine strains), the four genes *H*, *N*, *F*, and *P* were used, as was also the case for the Europe-2/European Wildlife, Europa 3/Arctic, Europe/South America-1, America-2, Asia-1, and Asia-4 (Figure 7B).

### 3.5. Genotype and Host Family

We have analyzed articles that reported the detection of different CDV genotypes in 14,667 carnivores from 76 species worldwide (Table 2). Most genotypes were identified in dogs, followed by wild canids, mustelids, felids, and finally procyonids (Figure 6A). Notably, the America-1 and Europe/South America-1 genotypes, followed by Asia-1, had the highest host species diversity among those considered to be the main hosts of CDV (i.e., domestic and wild canids, mustelids, procyonids, and felids).

Dogs were the species where the greatest genotypic variability was found (Figure 6A,B). With the Asia-3 exception, which was mainly sequenced from wild Felidae animals, all of the rest sequenced genotypes were found in dogs. This frequency pattern associated with dogs was observed, including rare genotypes sequenced as South America-3, Asia-2, Asia-4, and Asia-5, which were only sequenced in dogs (Figure 6B). However, Asia-3 is the only genotype that has not been detected in dogs, while it has been detected in wild Canidae and Felidae. In decreasing order, the genotypes most frequently isolated in dogs were Europe/South America-1 (23.8%), Europa-3/Arctic (22.5%), Asia-1 (15.6%), and America-1 (8.8%) (Figure 6B).

Europe/South America-1 (6.4%) was the most frequently sequenced genotype in wild Canidae (29.4%) and Mustelidae (45.5%) species. In wild Canidae, the next most detected genotypes were Europa-2/European Wildlife (16.2%), Asia-1 (11.8%), and Europa-3/Arctic (10.3%). On the other hand, in the family Mustelidae, the next most frequently reported genotypes were America-1 (18.2%), Europa-2/European Wildlife (15.9%), and Asia-1 (13.6%). Within the family Procyonidae, those genotypes that are more frequent in the Americas are predominant, such as America-1 (30.8%), America-2, America-4, Europe/South America-1 (all three represented with 15.4%), and Europa-2/European Wildlife (7.7%). Concerning other less represented Caniformia families, in the Ursidae, the genotypes Asia-1, Europe/and America-1 and America-1 have been detected.

With respect to the suborder Feliformia, the main genotypes isolated in wild Felidae were Africa-2 (27.8%), Asia-3 (22.2%), Europa-3/Arctic (16.7%), America-2, and Europe/South America-1 (both of them with 11.1%). In the family Hyaenidae, only the genotype Africa-2 has been detected, while in the family Viverridae, only the genotype America-1 has been detected. The families Mephitidae (Caniformia) and Herpestidae (Feliformia) have tested negative for CDV in several studies (Table 2).

### 3.6. Global Geographic Distribution of Genotypes, Hosts, and Studies

**Figure 8:**
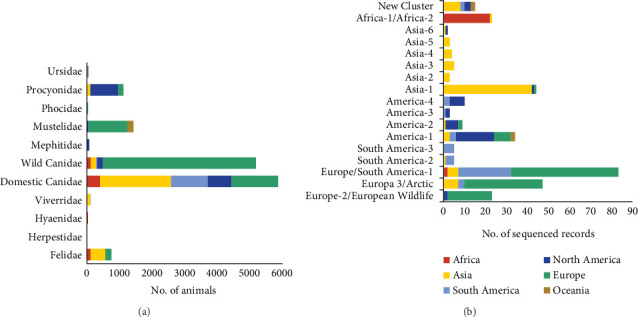
(A) Geographical distribution by continent and carnivore family of the animals studied in 457 analyzed records. (B) Geographical distribution by continent of the genotype in 332 sequenced records (records in which the CDV was sequenced). CDV, canine distemper virus.

Considering all carnivores studied according to their continent of origin, more than 70% of them were collected from Europe (52.37%) and Asia (21%), almost 21% in North (13%) and South America (8%), and the rest from Africa (4.5%) and Oceania (1.2%). Dogs (5868 animals) were the most frequently analyzed carnivore species, making domestic canids the only species that has been analyzed in all continents worldwide, with Oceania exception. The second most studied group, wild Canidae (5169 animals), was mostly studied in Europe (4694 animals; 90.5%), while the rest of them were studied across the rest of the continents. Felids were studied mainly in Asia (61.3%), Europe (22.3%), and Africa (14.6%), while procyonids were analyzed in North America (76.4%) and to a lesser extent in Europe (14.3%) and Asia (9.3%) (Figure 8A).

The analysis of the geographical distribution of the genotypes reported in each continent (Figure 8B) showed that among the genotypes with the highest frequency of sequencing, America-1, and Europe/South America-1 genotypes were the ones detected in the largest number of continents (although they do not follow the same territorial pattern), demonstrating their wide geographical distribution worldwide. Asia-1, America-2, and Europe-3/Arctic were also cosmopolitan lineages (being detected in at least three different continents). Finally, the New Cluster group, which included CDV lineages not yet classified into one of the 17 main genotypes, was reported from four geographical areas: Asia, Oceania, North, and South America (Figure 8B).

**Figure 9:**
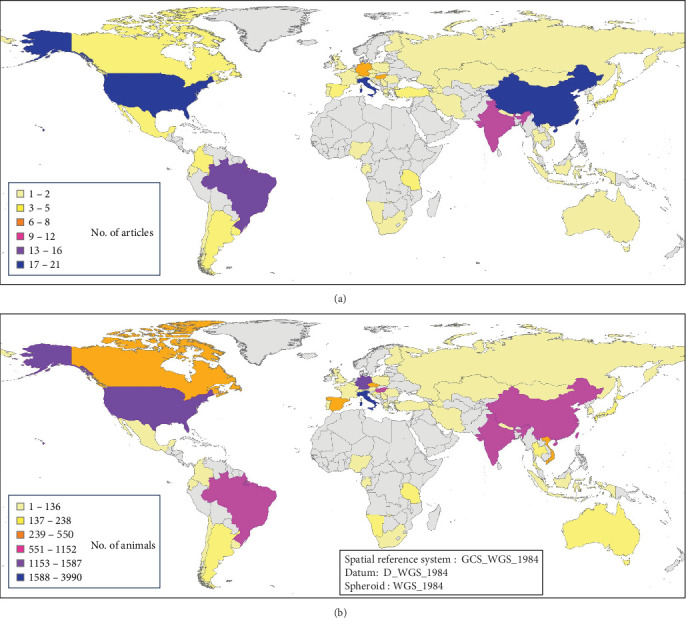
(A) Geographical distribution by country of the 160 analyzed articles. (B) Geographical distribution by country of the number of animals analyzed in 457 records from the 160 reviewed articles.

Taking into account the species studied and the pressure of CDV research and sequencing that has been done in the different parts of the world (Figure 9A,B) have analyzed the geographical distribution of articles in which CDV genotypes are sequenced and identified, as well as the number of animals studied in the published research. This geographical analysis showed differences between countries. Italy is one of the top countries in terms of the number of articles published and the number of animals analyzed, while Germany has a medium number of published articles, but researchers have made a very large effort with the sample size, as it appears to be one of the countries in the world with the highest number of animals with sequenced CDV, and also a very high number of animals studied. However, India has a higher publication effort, and also the number of different species analyzed is very high, although the total number of animals analyzed was lower. Countries such as the United States of America, China, Italy, and Brazil are at the top, both in terms of published articles with CDV sequences and the number of animals analyzed. In contrast, there are gaps in many countries in Africa, Oceania, Central and South America and the Baltic Sea area, where no CDV sequencing studies are reported.

**Figure 10:**
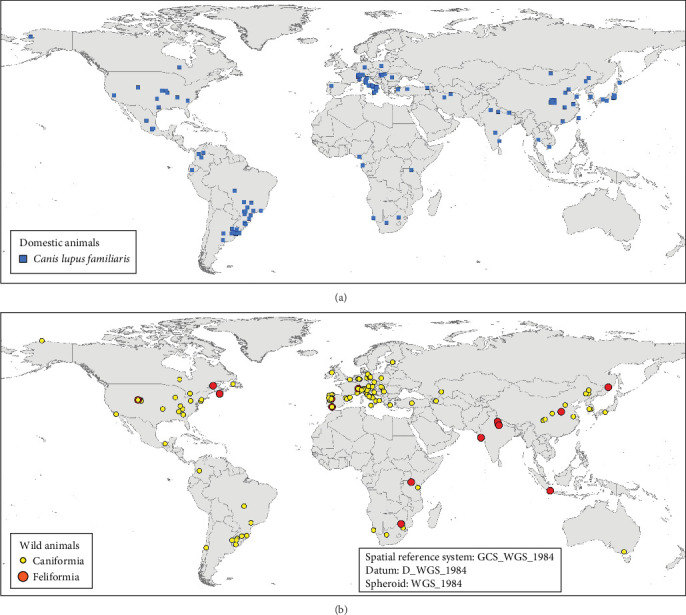
(A) Geographical distribution by country of the 5868 domestic dog species (*C. lupus familiaris*) studied in 457 records from the 160 reviewed articles. (B) Mapping by country of the 8799 wild animals, classified as Caniformia and Feliformia suborders, studied in the same records from the reviewed articles.

It is important to highlight that the dog was the most analyzed target species, being present in studies carried out all over the world (Figure 10A), and the global geographic distribution of the sequenced genotypes shows this. A similar analysis with the remaining nondog host species showed that there is a specular distribution in terms of global geographic distribution. The data analyzed during these 39 years of sequencing CDV genotypes showed, with exceptions, that CDV from wild hosts is sequenced in those regions where CDV is sequenced in dogs. The main areas from which the largest number of CDV genetic sequences in dogs originate have research teams that have also studied this pathogen in more depth in wild hosts. Considering all carnivorous dogs and nondogs studied according to their country of origin, most of them were collected in Italy, China, South America (Brazil and Argentina), and the United States (Figure 10 A,B).

### 3.7. Temporal Frame of the Main Genotypes

**Figure 11:**
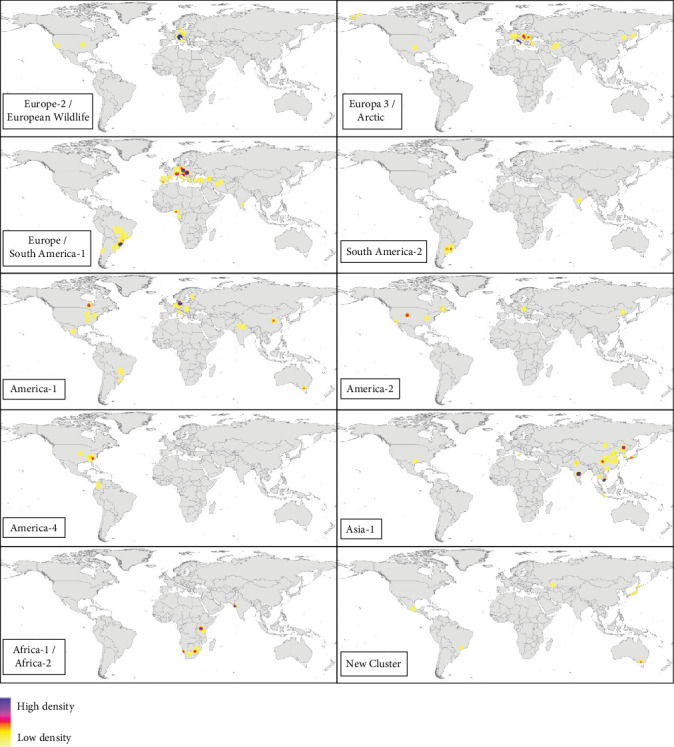
Geographical distribution of the main CDV genotypes sequenced between 1985 and 2024: **Europe-2/European Wildlife** (including Europe-2 genotypes); **Europe-3/Arctic** (including Arctic-like genotypes); **Europe/South America-1** (which includes genotypes reported as Europe-1 from 2001 (Martella et al. [52]) to 2024 (Huang et al. [53]) and genotypes reported as Europe/South America-1 from 2009 (Megid et al. [54]) to 2023 (Ndiana et al. [55]); **South America-2** (including South America 2 Argentina and other strains from Argentina); **America-1** (including Western vaccine, Rockborn like and North America-1 genotypes); **America-2**; **America-4** (including South America/North America-4); **Asia-1; Africa-1** (which includes Africa-1/Southern Africa genotypes); **Africa-2** (including Africa-2/Eastern Africa genotypes); **New Cluster** (which includes those genotypes that have not been classified into any of the 17 genotypes). The following genotypes could not be represented on world maps due to their local distribution area. Detailed information on these genotypes is given in Sections 3, and 4: **America-3** (including North America-3 genotypes); **South America-3** (including Colombia strains); **Asia-2**; **Asia-3**; **Asia-4**; **Asia-5**; **Asia-6**. CDV, canine distemper virus.

Regarding the genotypes isolated according to the country of origin, Figure 11 shows the cosmopolitan potential of CDV. The **Europe/South America-1** genotype was the most frequent worldwide, founded in Europe, South America, Asia (India, Turkey, Iran), and Africa (Gabon, Nigeria). This genotype consists of two major lineages, and it was mostly detected in the suborder Caniformia (80/83 records). On the one hand, the Europe-1 lineage (52/83 records), which was detected between 2001 and 2024 in Europe, both in dogs [52] and wild carnivores as European badgers [53]; on the other hand, the Europe/South America-1 lineage (31/83 records) which was reported between 2009 and 2023, mainly in South America, since 2006 in Hoary's foxes (*Lycalopex vetulus*) from Brazil [54]. Europe/South America-1 was also reported in dogs in Uruguay and Brazil [31, 60], and outside the Americas, it has been reported in 2023 in dogs in Nigeria [55], moreover in several carnivore species in Europe (Figures 8B and 11, Supporting Information 3). Europe-1 lineage was detected in 2002 in both European lynx species (suborder Feliformia), specifically in Switzerland in European lynx (*Lynx lynx*) [38], and in 2003 in Iberian lynx (*L. pardinus*) in Spain [61] (Supporting Information 3).

Another of the most frequent genotypes in Europe, in addition to the Europe-1 lineage, was the **Europe-3/Arctic** genotype (Figure 8B). This genotype was reported between 2003 and 2024 years, both in wild and domestic carnivores (mostly in the suborder Caniformia, 44/47 records), predominantly in Europe, but it was also detected in 2003 and 2012 in dogs in the USA [62, 63] and in 2004 in foxes (*V. vulpes*) in China [64] (Figure 11). Within the suborder Feliformia, it was detected between 2001 and 2010 in Siberia Amur Tiger (*Panthera tigris altaica*) in Russia [65] (Figure 11, Supporting Information 3). The **Europe-2/European Wildlife** genotype was mostly detected in Europe in several families of wild carnivores of the suborder Caniformia (Canidae, Mustelidae, Procyonidae, and Ursidae) (Figures 6B, 8B, and 11); however, it was detected in 2007 in dogs in the USA [66] (Supporting Information 3).

The genotype **America 1** was also one more cosmopolitan genotype, one of the most widespread geographically being reported in countries from North (USA and Canada) and South America (Mexico, Brazil), Europe (Italy), Asia (India), and Oceania (Australia). Moreover, this genotype was one of the oldest in terms of reports in the scientific literature between 1999 and 2024 (Figures 8B and 11; Supporting Information 3). Another genotype from the Americas continent is **America-2** (2.97%), which was first reported in dogs in 2005 in Hungary [67], and since then, it has been detected in wild Caniformia in the USA, such as Island fox (*Urocyon littoralis*) [68], raccoon and coyote (*Canis latrans*) [69], and in wild Feliformia (both species American lynx, *Lynx canadensis* and *Lynx rufus*) samples collected in 1989 in Canada [70] (Supporting Information 3).

Some genotypes were clearly restricted to the geographical area that gives them their name and possibly with a lower dispersal capacity, such as **America-3**, **America-4**, and South America-3 genotypes. America-3 (0.99%) and America-4 (3.3%) are genotypes that were reported in Central and South America, moreover in North America (Figure 11); both genotypes were only detected in the suborder Caniformia (Figure 6B). America-3 was detected in 2006 in dogs in Ecuador [71] and in 2013 and 2014 in raccoons [69] and dogs [72], respectively, in the USA. The America-4 genotype includes several lineages named South America/North America-4 and North America-4; the first records were from the USA in 2010 in dogs, red foxes (*V. vulpes*), and raccoons [73] and in 2013 in gray foxes (*Urocyon cinereoargenteus*) [18]. It was later detected in Colombia in 2017 in dogs [74] and in 2021 in Crab-eating foxes (*Cerdocyon thous*) [34] (Figure 11; Supporting Information 3).

Classically, South American genotypes are considered to be **South America-2** (1.65%) and **South America-3** (1.65%), which have only been reported in species belonging to the suborder Caniformia and mostly in dogs (Figure 6B). South America-2 includes the lineages detected in dogs [28, 75, 76] and in crab-eating foxes [77] in Argentina, in addition to a strain detected in 2019 in a dog in India [78] (Figure 11). The South America-3 genotype includes lineages detected in Colombia between 2011 and 2017 [60, 79] (Supporting Information 3).

Among the Asian genotypes, the **Asia-1** genotype was more cosmopolitan and reported than the other Asian genotypes (Figure 6B) and over a longer period (from 2007 to 2024) (Supporting Information 3). The vast majority of CDV detections were in families of the suborder Caniformia (43/44 records), such as Canidae, Mustelidae, Procyonidae, and Ursidae (Figure 6B). The only record of the suborder Feliformia occurred in 2022 in a leopard (*Panthera pardus melas*) in Indonesia [80]; moreover, it has been detected in 2008 in noncarnivorous animals (*Macaca mulatta*, family Cercopithecidae) in China [9]. Geographically, it is mostly found in Asian territories (43/44 records), except for one record in the USA, in a dog in 2008 [81] (Figure 11). China is the country with the most records (28/44), but it has also been detected in South Korea [82], Japan [83], Thailand [84] and recently in dogs in Vietnam in 2022 [85], India in 2023 (strains detected in 2015 reported by Agnihotri et al. [86], strains detected in 2018 reported by Dema et al. [87]) and Mongolia in 2024 [88] (Figure 11; Supporting Information 3).

The **Asia-2** genotype was scarcely reported (0.99%) and was detected in dogs in South Korea [82] and Japan [89] (Figure 6B). However, the **Asia-3** genotype (1.65%) was only detected in wild carnivores, mainly in species from the Feliformia suborder (4/5 records), ranging from red fox (*V. vulpes*) in China [64] to felids such as Asiatic lion (*P. leo persica*), leopard (*P. pardus*), tiger (*P. tigris*), and palm civet (*Paradoxurus hermaphroditus*) from India [45]. Other rare genotypes in Asia, such as **Asia-4** (1.32%) and Asia-5 (0.99%), are detected only in dogs (Figure 6B). Asia-4 was detected in 2010 in Thailand [84], 2011 in China [90], 2019 in India [78] to 2021 in Mongolia [88]. **Asia-5** was detected in India in 2015 [35] and in Nepal in 2018 [91]. As the last Asian genotype, **Asia-6** (0.66%) was detected in dogs in 2004 in the USA [92] and subsequently detected in lesser pandas (*Ailuropoda fulgens*) in 2018 in China [93] (Figure 6B; Supporting Information 3).

Finally, the genotypes mostly detected on the African continent were Africa-1 (3.3%) and Africa-2 (4.29%). Africa-1 includes the lineages detected in 2001 in dogs and black-backed jackals (*Lupulella mesomelas*) in Namibia [94] until the detection in 2018 in an Asiatic lion in India [95]. The Africa-2 genotype includes strains analyzed in Tanzania from 1993 to 1994 in dogs, African lions (*P. leo*), spotted hyenas (*Crocuta crocuta*) and bat-eared foxes (*Otocyon megalotis*) [96, 97], to the most recent analysis of strains in 2007 in African wild dogs (*Lycaon pictus*) and in 2011 in golden jackals (*Canis aureus*) [30] (Figure 11; Supporting Information 3).

## 4. Discussion

CDV was initially described in domestic dogs, but scientific evidence gathered in the last decades indicates that it should be approached as a global multihost pathogen of concern, given that it can affect a wide range of wild carnivores; but owing to their wide distribution and receptivity, domestic dogs continue to be considered the primary reservoir for CDV infection [15, 19]. Over the nearly 40 years covered by this scoping review, it has been shown that canids are the target hosts for this virus, as more than 90% of the animals from which CDV was isolated and sequenced in this scoping review belonged to the suborder Caniformia. Within this suborder, 40% of the studied animals were dogs, and the nondog species most represented belonged to wild Canidae, Mustelidae, and Procyonidae (especially red foxes, raccoons, and badgers); concerning Mustelidae, our results indicate that mustelids are particularly noteworthy, among which badger, European otter and polecat stand out. Within the suborder Feliformia, the family Felidae represents an important group susceptible to CDV, including the Asiatic lion and Iberian lynx.

The emergence and spread of CDV are currently of great interest to animal health authorities around the world. The evidence that the virus is a highly ubiquitous pathogen, affecting domestic and wild species that phylogenetically belong to different host families, suggests that CDV is a challenging and complex health problem to mitigate and control. In this sense, Zhao et al. [64] experimentally demonstrated the high sensitivity and affinity of the virus for red foxes and raccoons. The high representation of foxes with CDV lineage sequences (30.23%) observed in this long-term review showed that this species might play a key role in the spread of CDV due to their high ecological plasticity to diverse habitats and wide spatial distribution in the world. The seroprevalence of CDV in fox populations from the western United States was 13% [98], while data from Europe show varying rates of 4%–30.5% in countries such as Spain, Portugal, Italy, and Germany [79]. In the case of raccoons, our study indicates that 7.23% of the samples analyzed worldwide have been CDV positive and sequenced; in a similar way to the red fox, this procyonid has a great capacity to adapt to different environments, including natural habitats, anthropized rural and even urban areas. In their native North American habitat, raccoons are known to respond positively to urban environments with high human population densities [99, 100]. Therefore, the epidemiological relevance of this species can be very high, as demonstrated in the United States, where a mortality rate of 45% caused by CDV was reported in free-living raccoons found inside, around, and outside a zoo in 2001 [14].

Our study points out that CDV isolation and sequencing in carnivores belonging to the Feliformia suborder have been performed in far fewer animals (6%) than in the case of Caniformia host species. In fact, CD is not a clinically recognized entity in domestic cats; however, large felids are susceptible to infection with CDV. Most of the large felids are threatened or endangered species; thus, surveillance of pathogens that have the potential to cause their extinctions is critical [4, 45]. In this sense, the first articles showing interest in sequencing CDV strains detected in wild felids date back to the CDV epidemic emerged in the Serengeti National Park in Tanzania in November 1993 [96, 101] and then spread through the Serengeti ecosystem causing high morbidity and mortality in juvenile and adult African lions (*P. leo*) and spotted hyenas (*C. crocuta*) [30, 102, 103]. Recent studies in India [45, 95], China [104], and Indonesia [80] show the detection and prevalence of CDV in wild felids and viverrids of the Asian continent, sequencing several CDV lineages in Asiatic lions, tigers (*P. tigris*), leopard (*P. pardus* and *P. pardus melas*), palm civet cat (*P. hermaphroditus*), and masked palm civet (*Paguma larvata*), among others, demonstrating the importance of these hosts in the CDV spread and questioning the role this virus may play in the survival of these endangered wild populations. In the present review, the Iberian lynx and the Asiatic lion, which are considered two of the most endangered felid species in the world [105, 106], were among the carnivore species in which CDV was most frequently sequenced, demonstrating the concern about such a threat to the conservation of these species.

When studying wild animals, it is essential to have quality biological samples for etiological diagnosis, especially if the genetic material detected is to be sequenced. We analyzed the number of samples and the type of biological material used for CDV detection in the reviewed studies. Dog was the species with the highest number of samples collected worldwide to detect CDV, which was to be expected considering that it is the main host species of this virus. Concerning the other species of the suborder Caniformia, the highest number of samples was found in those wild carnivores that are more sensitive to the CDV virus, such as canids and mustelids. It is striking that procyonids, traditionally considered very sensitive to CDV, did not contribute with a high number of samples analyzed over the almost 40 years studied; however, despite not being one of the most analyzed, the raccoon has more CDV sequences than the badgers. Other carnivores from the Mustelidae family, including ferrets (*M. putorius furo*), have been models for CDV infection in experimental studies [107, 108]. The fact that mustelids are second only to dogs, both in number and in the variability of sample type analyzed to detect CDV, may reflect the revealing the sensitivity of this species to CDV [109, 110], the economic importance (farmed for their fur) of some mustelid species such as martens (*Martes* spp.) and minks (*Mustela* spp.), their role as semi-domestic animals and, in the case of badgers, their social and gregarious behavior and their presence at the domestic–wildlife interface, which facilitates their study in comparison with other species in the order Carnivora that are more elusive. All these characteristics, together with their abundance, make mustelids easier to sample than other carnivores.

The type of biological sample used for CDV detection and sequencing studies corresponded mainly to those target organs in the pathogenesis of CDV. In this sense, the brain, lung, and spleen are considered replication sites and suitable tissues for CDV detection by PCR for both domestic and wild carnivores [12, 20]. On the other hand, biological samples extracted from live animals, such as nasal and conjunctival discharges, urine, and feces, correspond to CDV entry and excretion routes in the acute phase of clinical disease [111]. Our study indicates that organs from carcasses were more easily collected from wild carnivores, found dead, road-killed, or hunted. On the contrary, secretion and excretion samples were collected from live carnivores, corresponding with domestic dogs. The importance of obtaining all types of biological samples when working with wildlife is evident in the case of mustelids. In this regard, mustelids have been involved, together with red foxes, in several CDV outbreaks during the last decade affecting Northern Italy [41, 43], Southern Italy [112], South Bavaria [113], and Switzerland [38]; to study these outbreaks, pools from a wide variety of organs were used, including brain, lung, stomach, intestine, conjunctival, nasal and rectal swabs, urine (or swab from bladder) and intracardiac clot [12, 43], showing how important it is in studies involving wild carnivores to get several types of samples for detecting CDV and reducing the number of false negative animals.

This review evaluated the different laboratory techniques that have been used over 39 years for the detection of CDV in carnivores. The families with the highest number of laboratory analyses corresponded again to those species that are most sensitive to CDV: dogs, wild canids, mustelids, and procyonids. In addition to the detection and sequencing of CDV by PCR techniques, the studies analyzed in this review performed complementary techniques (serology, histology, and IHC, that allow direct and/or indirect detection of CDV from serum and tissues). The use of these complementary techniques can be a less expensive and simpler way for a first approach to the diagnosis of CDV, to be followed by molecular detection and sequencing by PCR. Several studies diagnosed CDV by enzyme-linked immunoassay (ELISA) designated for use in domestic dogs [114, 115]. ELISA was used to detect IgM and IgG antibodies against CDV in domestic dogs and other carnivore species. However, the use of serological techniques in wild carnivores requires adaptation, as species-specific secondary antibodies for wildlife are not commonly available [116].

The dog is clearly the species in which CDV has been studied the most globally, being likewise the most numerous Caniformia species in the world, with an estimated population of over 700 million worldwide [117]. Domestic dogs are considered to be the primary reservoir of CDV and, therefore, a key epidemiological factor in the spread of the virus between free-ranging, unvaccinated, or incompletely vaccinated dogs (pets and feral individuals) and, in addition, to urban and rural wildlife [3]. Thus, interactions between domestic canids and wildlife result in CDV spillover phenomena and have been shown to trigger high mortality in wildlife species. Also, CDV spill-back from wildlife to domestic canids is possible [14, 33]. However, little is known about CDV in wild carnivore populations that have direct and regular contact with domestic dogs [3]. Therefore, the use of molecular and sequencing techniques helps us to understand the interactions and pathways of pathogen transmission between potential hosts at the domestic–wild interface. Viral isolation and genomic sequence analysis are powerful techniques that can be used to track viruses from host to host. Studies of amino acid substitution in CDV strains from ecosystems where there is interaction between wild and domestic canid and noncanid species help to understand the adaptation of CDV to different hosts and its expansion potential [30]. The development of molecular techniques brings diagnostic tools that are highly sensitive and specific [24]. One of the techniques that have been developed worldwide for the detection of CDV is the RT-PCR assay [19]. However, the technique most widely used by researchers for CDV detection has been real-time RT-PCR, a more rapid diagnostic technique for the detection of CDV which is used for both diagnostics and research and is especially useful for pathogen detection. Additionally, nested PCR techniques have been developed for the detection of CDV [118].

Concerning the host species analyzed in this 39-year review, dogs, wild canids, mustelids, wild felids, and procyonids were the most frequent carnivore families to be subjected to CDV PCR detection and subsequent sequencing. In addition, like dogs, mustelid samples have been used in a wide range of analyses and with a wide variety of PCR techniques. Most genotypes have been detected in domestic dogs and, later, in wild canids, mustelids, and procyonids. From an epidemiological point of view, this finding may mean that mustelids, wild canids, and procyonids are (i) more prone to contact with domestic dogs (considering these as primary hosts of CDV) since they coexist more easily at the domestic–wild interface [119], and (ii) the CDV appears in species with gregarious social behavior, which due to its high transmission capacity, as is the case with most representatives of the genus Morbillivirus [120], facilitates the intraspecific transmission. Among wild canids, foxes and wolves are the species on which most studies have been reported.

Different CDV genotypes have been detected in wolves in several European and American countries, in jackals in Africa [30, 94] and Europe [37, 121], in maned wolves in South America [122], and coyotes in North America [69, 123]. In relation to the wolf populations in Europe, serological data suggest diffuse immunity at the population level [124], and the Alpine wolf population has been under-represented in the CDV mortality events observed in this area since 2006 [125, 126]. Despite this, it is relatively common to detect CDV in wolves with nervous signs, and CDV outbreaks in this species are considered to be evolutionary factors in well-studied populations in North America [127, 128]. The Europa 3/Arctic-like and Europe-2/European Wildlife lineages detected in wolves in Spain [114] and Italy [12, 115], which are typical of domestic dogs, highlight the domestic origin of the infection, showing spillover processes in areas shared by wild and domestic canids [129]. In the same vein, viral strains have been detected in wild carnivores (foxes and mustelids) in the Alps, which have adapted to these wild hosts, being grouped in the European Wildlife genotype but which, recently, have been involved in spillover episodes detected in dogs [126].

Regarding the foxes as CDV hosts, recent studies show the high susceptibility of this species to CDV [64, 116]. This susceptibility to CDV makes this canid one of the world's major wild reservoirs of this virus. In Europe, the number of genotypes detected in foxes from 1996 in Germany [109, 116] to 2024 in Spain [53] is very high. The genotypes most frequently detected in this species are Europe/South America-1 (from 2000 [52] to 2024 [53]) and Europe-2/European Wildlife (from 2011 [41] to 2022 [130]). To a lesser extent, they also detect Europe-3/Arctic [64], America-1 [109, 123], America-2 [131], Asia-1 [64, 83], and Asia-3 [64]. We can assume that the wide global distribution and generalist behavior of foxes make them an essential vehicle for the spread of different CDV genotypes and, thus, an appropriate sentinel species.

As noted above, Mustelidae was the carnivore family in which the highest number of CDV PCR analyses were performed (Figure 5A); within this family, the highest number of samples and sequenced viruses were from badgers, a species in which CDV detection may be correlated with a higher probability of contact with domestic and wild canids at the domestic–wild interface and with the species' susceptibility to CDV virus. Another species highlighted in the CDV epidemiology is the raccoon, which is a generalist species that tends to inhabit the domestic–wild interface, where contact with domestic dogs is more likely [99, 132] and where they are considered a source of CDV infection [18, 69, 133]. The results of this study show that Procyonidae is one of the wild carnivore families in which CDV has been detected most frequently, including the American genotypes as America-1 [134], America-2 [135], America-3 [69], America-4 [18, 73]. To a lesser extent, the following have been detected: Europe/South America-1 and Europe-2/European Wildlife in Europe (Germany [100, 136], Czech Republic [47]), and Asia-1 [83]. The detection of these genotypes is a consequence of the areas of origin of raccoons (North America) and the current distribution areas in which this procionid is an introduced exotic animal (Asia and Europe). In the latter areas, it has been observed that the raccoon, by integrating with the autochthonous community of carnivores, assumes an epidemiological role in the spread of the multihost CDV genotypes present in these areas that colonize [100].

Direct contact (understood in wildlife as contact at less than 10 m distance [137]) with an infected individual is necessary for the CDV to spread, so the transmission rate is, theoretically, higher in gregarious species. For example, if the species is primarily solitary, such as tigers and leopards, infection usually occurs by the introduction of the virus from another species, and a key route of transmission is predation on roaming dogs or wild mesocarnivores, such as foxes [138]. This pattern may explain the increased detection of CDV in big felines, such as tigers, leopards, and lions (*Panthera* spp.), in Asia [45, 95, 139] and Africa [96, 101, 102]. In this regard, it should be noted that, although the dog is the main CVD host, this multihost pathogen can cause constant spillover phenomena in which several species of the suborder Feliformia may be involved; this situation has served as an argument for researchers to advocate for a renaming of this virus, seeking a name that more accurately reflects its multihost potential character [140].

Regarding the genomic and molecular aspects included in this review, we can highlight that nucleic acid sequence analysis of the *H* gene is the gold standard for phylogenetic analysis, classification, and genotyping of CDV because it has the greatest heterogeneity of the six structural proteins of CDV [3]. Considering that our scoping review provides a historical overview of CDV sequencing, spanning from 1995 [27, 96] to 2024, although WGS is now commonly used, we include data from studies using multiple genes to reflect the historical evolution of sequencing techniques. Thus, gene *H* was the most widely used gene for sequencing this pathogen in carnivore hosts. H protein possesses a high percentage of mutations that undergo positive selection to adapt to its new host [6], and specific amino acid substitutions within the H protein are involved in spillover events and are thought to have contributed to the spread of CDV from dog to other mammal carnivore species [15, 19]. The specificity with which the CDV *H* gene interacts with signaling lymphocytic activation molecule (SLAM) receptors and its potential as a determinant of host range has been investigated [19], probably enabling the natural infection of noncanid hosts, as the outbreaks in breeding colonies of rhesus macaques (*M. mulatta*) and cynomolgus macaques (*Macaca fascicularis*) have raised several concerns of a potential zoonotic risk of CDV in humans [15]. This high mutational capacity of the *H* gene makes it a high-capacity tool to differentiate lineages of wild-type CDV for molecular epidemiology studies [141, 142].

Other less frequently used genes for CDV amplification and sequencing by PCR are the *N*, *P*, and *F* genes. The *F* and *P* genes, which are considered to be highly conserved parts of the CDV genome, are often used in combined and hemi-nested PCR when distinguishing between wild-type CDV strains and vaccine strains or when mixed with wild-type strains [141, 143, 144]. Concerning the FSP fragment, it is currently recognized as a valuable tool for genotype classification due to its high phylogenetic diversity. However, given the variability in the use of the fragment over time, we have simplified our analysis by classifying all studies using the FSP fragment into the broader *F* gene category.

Nowadays, CDV is one of the most cosmopolitan and globally spreading pathogens due not only to its high mutation potential, which has allowed it to grow from 6 major lineages in 2007 [142] to more than 19 in 2024 [10] but also to its ability to jump between species. These lineages are defined according to the amino acid divergence of the H protein between the different strains [79]. In this sense, Martella et al. [142 ] sequenced some CDV Italian strains, showing that intralineage variation within the major European cluster was <3.5% amino acids, while variation from the other lineages was >4%. Moreover, the cocirculation of different lineages in the same habitat may result in homologous recombination and the emergence of new viral strains or sublineages [6]. The possibility of recombination of old vaccine strains with new viral variants has been suggested as the reason for the evolution of CDV and disease in vaccinated animals [120]. Therefore, CDV taxonomy is continuously evolving as detection and sequencing studies are conducted on more species from various regions around the world.

Analysis of CDV strains identified in various geographical areas and from several carnivore species revealed that the genetic/antigenic drift acting on specific structural proteins of CDV is driven mainly by a geographical pattern. Accordingly, the present review has identified 17 major lineages that account for the majority of the CDV strains detected in the field. About the genotype most identified worldwide, Europa/South America-1, it is necessary to clarify that this genotype is made up of two major lineages (Europa-1 and Europa/South America-1), which have been reported to be named interchangeably in the scientific literature, although there is now agreement to call it Europa/South America-1 [76]. Those genotypes reported as Europe-1 have been detected from 2001, in a red fox strain identified in Italy [52] to badger (*M. meles*) strains identified in Spain in 2024 [53]. On the other hand, genotypes reported as Europe/South America-1 range from the 2009 citation [54] to 2023, with a dog citation in Nigeria [55]. The oldest European/South American-1 strain was detected in 1997 in several mustelid species in Hungary, although it was not sequenced until 2022 [55]. In South America, the oldest sequenced strains were detected in 2006 in dogs from Brazil, Uruguay, and Ecuador [31]. Other frequently identified and sequenced main genotypes were Europe-3/Arctic, followed by Asia-1, America-1, and Europe-2/European wildlife.

Concerning hosts, the dog was the species with the highest genotype diversity, with Europe/South America-1 and Europe-3/Arctic being the most represented, followed by Asia-1 and America-1. Among wild hosts, wild canids and mustelids had the highest genotype diversity, although we can speculate that there is a different host pattern for the different genotypes. In Europe, the most frequent genotype is Europe/South America-1, detected in wild canids and mustelids, whereas in Asia, the main identified genotype is Asia-1, detected in wild Canidae, Felidae, and Ursidae families. In addition, mustelids also showed high frequencies of Europe/South America-1, America-1, Europe-2/European wildlife, and Asia-1 genotypes. The diversity of families that form the domestic–wild interface on each continent may explain these differences [145, 146].

The most diversified genotypes in terms of carnivore families are undoubtedly Europe/South America-1, and the vaccine strains included in America-1, as they can be found in most of the main hosts of CDV: domestic and wild canids, mustelids, procyonids, and felids. Noteworthy, America-1 is the origin of most vaccine strains used in the West [6]. In addition to being the most host-diversified genotype, Europe/South America-1 is, after America-1, the most cosmopolitan genotype we have detected in this review of 160 articles.

In the geographical area of Europe, two different lineages were detected in an outbreak of CDV affecting the wild carnivore population of Northern Italy [38, 43, 126]. Since 2004, both Europe-2 and European Wildlife clustered together with viruses isolated from Hungarian dogs [41, 115]; thus, to facilitate epidemiological descriptions, the subgroup insight into the Europe-1 (now called Europe/South America-1) lineage formed by the Italian and Bavarian wild CDVs and the Hungarian strain is now termed as Europe-2/European wildlife [41]. This lineage proved to be well-adapted to wildlife, considering it was detected in wild carnivores for 12 consecutive years in Italy, as well as in other European countries [41]. In the records analyzed in this review, Europe-2/European wildlife was the lineage in which dogs are least proportionally represented, while it was isolated in wild canids, mustelids, procyonids, and ursids. The first citations in the literature were in 2008 in the USA [66], although the oldest sequenced strain was from 2006 in a study in Italy in marten, badger, and red fox [41]. The most recent CDV strains were detected in 2021 in brown bears (*Ursus arctos marsicanus*), dogs, and red foxes in Italy [130].

The Europe-3/Arctic lineage is mostly detected in Europe, although it is in the process of spreading to China, where it has been detected in foxes [64], and Russia, where it has been detected in the endangered Amur tigers (*P. tigris altaica*) [65], demonstrating the high potential for CDV spread due to its ability to find receptive hosts [115]. It is a lineage frequently detected in dogs but also in wild canids such as arctic foxes [63], golden jackals [121], and gray wolves [115]. The genotype is called Arctic because it was first detected in the late 1980s, when epizootics with high mortality were observed in seals in northern Europe and Siberia [147, 148], and the first sequence was published in dogs in Alaska in 2003 [62].

Africa-2 genotype caused an outbreak of CDV in the African lion, spotted hyena, and African wild dog (*L. pictus*) populations in Serengeti National Park [30, 96, 102]. Since then, this genotype has been isolated and sequenced in populations of these wild carnivores in Kenya and Tanzania, as well as in feral domestic dogs [97, 149, 150]. The mobility of CDV is evident, as there are CDV strains that have been subsequently sequenced in geographic locations far from their place of origin. This is the case of the 2018 outbreak of CDV in Asiatic lions in India, which was attributed to a genotype originating from southern Africa (Africa-1) [95]. In this regard, there is a human route between East Africa (Kenya, Tanzania, Madagascar, Mauritius, and India), which explains what happened with the spread of the virus from East Africa to South Asia: the Chinkunguya virus, which spread from an outbreak in Kenya in 2004 to several islands in the Indian Ocean and India, including Sri Lanka, Thailand and Malaysia [151]. In this sense, the African strains of CDV detected in East African lions may have followed a similar migration route to that of the Chikungunya virus.

Concerning Asia 1 to Asia-6 genotypes, our review examined articles that have sequenced in dogs and wild hosts belonging to the families Canidae, Felidae, Mustelidae, Procyonidae, and Ursidae. These studies show an ever-changing genotype landscape, confirming the mobility of CDV lineages along their host pathways. For example, the Europe/South America-1 [152] and Africa-1 [95] genotypes have been detected in wild hosts in the Indian subcontinent. The Asia-1 genotype is the most frequently detected in Asia. Forty-four records have been detected of which more than 90% are detected in strains from Asia, while one of them is detected in dogs from Texas (USA [81]). The latest detected strains of Asia-1 have been in dogs from Vietnam [85] and Mongolia [88], with dog strains of 2023 and 2024.

The Asian genotypes Asia-2, Asia-4, and Asia-5 have been detected only in dogs and have so far no records in wild Carnivora, whereas the Asia-3 and Asia-6 genotypes have only been detected in wild Carnivora and are frequent in large wild felids, thus becoming an additional risk factor that increases the possibility of extinction of endangered populations [45]. The Asia-2 genotype was detected between 2005 and 2008 in South Korea [82] and Japan [89]. The Asia-4 genotype was detected between 2013 and 2024 in Thailand [84], China [90], India [78], and Mongolia [88]. The Asia-2 genotype was detected between 2019 and 2023 in India, where they propose to change the name to India-1 [35] and Nepal. The Asia-5 genotype is derived from the Africa-2 genotype, the African genotype responsible for the outbreak in the Serengeti National Park, and given the very high similarity between Asia-5 and Africa-2, they can be considered “sister” genotypes [91]. As we can see over the years covered by this review, the worldwide distribution of genotypes showed that most are isolated and sequenced in the continent that gives them their name. However, CDV is constantly expanding, using the movements and introductions of its main hosts to modify the global distribution of its main genotypes. In 2005, the phylogenetic characterization of CDV detected in dogs in the USA showed similarity to CDV strains belonging to Asia-6 detected in a giant panda (*Ailuropoda melanoleuca*) from China [92].

Immunization with attenuated vaccines, belonging to the America-1 lineage, has been widely used for preventing CDV, considerably reducing its incidence in dogs [142, 153]. However, different publications have shown the presence of the vaccine strain in wild carnivores. These outbreaks might be a consequence of the emergence of new field strains able to avoid the immune response generated by the “old strains” currently used in the vaccines and/or because of the capacity of new field strains to infect other carnivore hosts involved in spillover events [25, 154–156]. This could explain the worldwide increase in the incidence of CD, even in vaccinated dogs, and outbreaks of CDV in wildlife [75, 157]. Currently, most CDV commercial vaccines in the United States, Canada, and Europe are formulated with strains still belonging to the America-1 lineage [73]. The increasing use of attenuated vaccines derived from America-1 is, in our opinion, a possible reason for the worldwide distribution of this genotype, as shown by several studies sequencing America-1, which turned out to be the most cosmopolitan genotype with the largest multihost community of all those detected in this review. In fact, America-1 has been sequenced in all the continents mentioned in these studies (America, Europe, South and North America, Asia, and Oceania), except for Africa. Interestingly, in 2019, America-1 was detected and sequenced for the first time in Asia in a captive population of masked palm civets [104], showing that the global pathway of America-1 through vaccines is continually spreading. In the case of the genotypes America-2, America-3, and America-4, their distribution areas are currently restricted to North America, where they are predominantly detected in wild hosts, mainly belonging to the families Canidae and Procyonidae. In addition, America-2 has been detected in wild felids, unlike the others in this group [70].

The distribution maps of the carnivore groups analyzed in CDV detection and sequencing studies showed that dogs were globally distributed and analyzed on all continents. Followed by domestic dogs, carnivores belonging to Caniformia depicted a worldwide distribution pattern that could indicate that the species of this group were epidemiologically the “wild mirror” of the CDV cycle. The mirror distribution suggests some issues to consider. On the one hand, it shows that in those areas (or study countries) where the greatest effort has been made to study CDV in dogs, there are also research teams where the greatest effort has been made to study CDV in wildlife. On the other hand, it also suggests spillover phenomena, which can occur from the dog to wild CDV reservoirs through predation on them [158] or through the use of shared habitats [102]. However, dogs are not the only source of infection in the domestic–wild cycle because spillover from wild mustelids to big felids has also been described [138]. In addition, spill-back phenomena have also been reported in recent literature, where wild genotypes have been sequenced in domestic dogs [91].

The constantly revised phylogeny of the CDV and the increasing analysis of wild species suggest a surprising and changing scenario involving this widespread multihost virus. Countries such as Italy, Brazil, the United States, China, and, in the last 3 years, India, led the way in terms of both the number of articles published with CDV sequences and the number of animals used for detection. In contrast, there are large data gaps in Africa, Oceania, and the Baltic countries, where no CDV sequencing studies have been reported. Accordingly, it would be advisable to assess the epidemiological status of domestic and wild carnivore populations in these regions of the world.

Finally, after an exhaustive study of the articles included in this review and others to support our discussion, we have confirmed the wide potential for the worldwide spread of the CDV and its high capacity to infect a wide range of host species within and outside the Carnivora order. However, we can also highlight that one of the shortcomings of our analysis was the impossibility of carrying out a temporal study of the appearance of the different genotypes in diverse host species and different geographical areas. Although it would have been interesting to analyze the variations of the lineages of each host population over time, the scarce number of articles performed on some of the studied species could have made it difficult.

### 4.1. Biases and Limitations of This Review

Language Restrictions: One of the limitations of this scoping review is the restricted language search, which was limited to Spanish, English, and Portuguese. While these languages represent a significant portion of scientific output, they do not encompass the entirety of research, excluding studies in Mandarin, German, French, and other languages. Furthermore, full-text access to many articles was unavailable.

Genotype Nomenclature Variability: The inconsistent naming of genotypes over time presented challenges in visually representing the prevalence of detected genotypes on maps. Specifically, the Europe-1 genotype was reclassified as Europe/South-America-1 by Megid et al. [54]. To address this, we decided to categorize all genotypes reported as either Europe-1 (from Martella et al. [52] to Huang et al. [53]) or Europe/South-America-1 (from Megid et al. [54] to Ndiana et al. [55]) from 2001 to 2024 under the broader category of Europe/South America-1.

Sequencing Depth and Quality: Our initial quality criterion required researchers to sequence a fragment of the detected CDV virus and attempt to classify it within a known lineage. We did not conduct a quantitative analysis of sequencing depth or perform a risk assessment, as our focus was on qualitatively analyzing the historical use of various genes for sequencing and only have as a criterion for classification that this sequencing has taken place.

Historical Perspective on Sequencing: Our scoping review provides a historical overview of CDV sequencing, spanning from 1995 [27, 96] to 2024. Although WGS is now commonly used, we included data from studies utilizing various genes to reflect the historical evolution of sequencing techniques.

FSP Fragment and Genotype Classification: The FSP fragment has been recognized as a valuable tool for genotype classification due to its high phylogenetic diversity. However, given the variability in fragment usage over time, we simplified the analysis by categorizing all studies using the FSP fragment under the broader category of the *F* gene.

Focus on Carnivora: Our review primarily focused on the Carnivora order due to the limited availability of CDV sequences from other orders in GenBank. While we identified studies on CDV in *Cercopithecidae*, *Myrmecophagidae*, *Hystricidae*, *and Sciuridae*, the overall focus remains on carnivores. Wang et al. [13] have recently sequenced the complete genome of a CDV strain (classified in the Arctic lineage) detected in 2018 in China and belonging to a species of the order Artiodactyla (*Sus scrofa*). The fact that there are few detections of CDV in species of orders other than Carnivora should not reduce the importance of these isolates, and these noncarnivore cases highlight the virus's ability to cross species barriers and its high mutation rate.

## 5. Conclusions

• We have shown the wide potential for the worldwide spread of the CDV and its high capacity to infect a wide range of hosts within and outside the Carnivora order.
• CDV shows a wide host diversity, with the dog (*C. lupus familiaris*) as the most frequent host and wild species of the family Canidae as the main wild host, with the red fox (*Vulves vulpes*) as the most significant species. The importance of wild secondary hosts differs according to the geographical area of the world, with Mustelidae and Procyonidae being the families involved in the maintenance of CDV.
• Europe/South America-1 and America-1 are the most cosmopolitan and diversified genotypes in terms of world distribution and carnivore families, which have been found in the majority of the main CDV host species: domestic and wild canids, mustelids, procyonids, and felids.
• Most of the lineages can be detected in several wild host families, in addition to the dog, suggesting constant spillover phenomena in shared habitats at the domestic–wild interface.
• Intercontinental migration of CDV lineages (such as America-1, Europe/South America-1, South America, and Africa 1 and 2) is observed, following the distribution routes of their hosts, showing that it is difficult to establish a fixed picture of this virus with high mutation potential, multihost capacity and in a globalized world.
• The high level of interest in CDV detection and sequencing reflects both its cosmopolitan nature and its high potential for genetic modification, as well as the need to modify its taxonomic classification, adapting it to the lineages detected in new geographical areas of origin and to the species carrying these genotypes.

## Figures and Tables

**Table 1:** Analyzed variables from the selected articles.

Category	Variable	Factor label
Host species	Wild or domestic carnivore	Wild carnivore; domestic carnivore
	Suborder	Caniformia; Feliformia
	Family	Canidae, Mephitidae, Mustelidae, Phocidae, Procyonidae, Ursidae, Felidae, Herpestidae, Hyanidae, Viverridae. Other families from orders such as Rodentia, Primates, Artiodactyla, and Proboscidea were excluded from the analysis but are included in Figure 1, Table 2, and Section 4
	Species	Common name
	Species	Scientific name

Analytical methods employed for CDV detection	Samples used	Blood, brain, lung, spleen, liver, lymphatic nodules, digestive system, kidney, bladder, ocular swabs, nasal swabs, urine, feces, others
	PCR methodology	The different types of PCR for detecting CDV have been classified into RT-PCR, RT-qPCR, nested-PCR, heminested PCR, combined PCR
	Other diagnostic methods	The different diagnostic techniques used for detecting CDV have been classified into serology, histology, IHC (immunohistochemical methods), and other methods employed
	Main genes used	Gene *H* (hemagglutinin), gene *F* (fusion protein), gene *N* (nucleocapsid), gene *P* (phosphoprotein), gen *M* (matrix), genes *V* and *C* (V and C proteins), gene *L* (RNA polymerase)

CDV lineages	Genotype (following [14] and [33])	**America-1** (including Western vaccine, Rockborn like, and North America-1 genotypes); **America-2**; **America-3** (including North America-3 genotypes); **America-4** (including South America/North America-4); **Europe/South America-1** (which includes genotypes reported as Europe-1 from 2001 [52] to 2024 [53] and genotypes reported as Europe/South America-1 from 2009 [54] to 2023 [55]; **South America-2** (including South America 2 Argentina and other strains from Argentina); **South America-3** (including Colombia strains); **Europe-2/European Wildlife** (including Europe-2 genotypes); **Europe-3/Arctic** (including Arctic-like genotypes); **Asia-1**; **Asia-2**; **Asia-3**; **Asia-4**; **Asia-5**; **Asia-6**; **Africa-1** (which includes Africa-1/Southern Africa genotypes); **Africa-2** (including Africa-2/Eastern Africa genotypes); **New Cluster** (including those genotypes that have not been classified into any of the 17 genotypes listed above)

Time and geographical data	Year of sampling	1988–2024
	Continent	Africa; America; Asia; Europe; Oceania
	Country	—
	Geographical region	—
	Latitude coordinates (decimal degrees)	—
	Longitude coordinates (decimal degrees)	—
	Accuracy	Accurate; Approximate; National

*Note*: Bold letters to highlight the CDV lineages.

Abbreviation: CDV, canine distemper virus.

**Table 2:** Number (*N*) and percentage (%) of animals from the order Carnivora analyzed in the 160 scientific articles reviewed.

Suborder	*N*	%	Family	*N*	%	Species		*N*	%	PCR
Feliformia	891	6.07	Felidae	753	5.13	African lion	*P. leo*	104	0.71	PCR +
	—	—	—	—	—	Asiatic lion	*P. leo persica*	329	2.24	PCR +
	—	—	—	—	—	Jaguar	*Panthera onca*	1	0.01	PCR +
	—	—	—	—	—	Snow leopard	*Panthera uncia*	2	0.01	PCR +
	—	—	—	—	—	Tiger	*P. tigris*	20	0.14	PCR +
	—	—	—	—	—	Siberia Amur tiger	*P. tigris altaica*	5	0.03	PCR +
	—	—	—	—	—	Leopard	*P. pardus*	91	0.62	PCR +
	—	—	—	—	—	Bobcat	*L. rufus*	5	0.03	PCR +
	—	—	—	—	—	Canada lynx	*L. canadensis*	5	0.03	PCR +
	—	—	—	—	—	Eurasian lynx	*L. lynx*	1	0.01	PCR +
	—	—	—	—	—	Iberian lynx	*L. pardinus*	165	1.12	PCR +
	—	—	—	—	—	Cheetah	*A. jubatus*	2	0.01	PCR -
	—	—	—	—	—	Clouded leopard	*Neofelis nebulosa*	1	0.01	PCR +
	—	—	—	—	—	European wild cat	*F. silvestris*	2	0.01	PCR -
	—	—	—	—	—	Fishing cat	*Prionailurus viverrinus*	3	0.02	PCR +
	—	—	—	—	—	Leopard cat	*Prionailurus bengalensis*	4	0.03	PCR +
	—	—	—	—	—	Jungle cat	*Felis chaos*	5	0.03	PCR +
	—	—	—	—	—	Puma	*P. concolor*	3	0.02	PCR -
	—	—	—	—	—	Palm civet cat	*P. hermaphroditus*	3	0.02	PCR +
	—	—	—	—	—	Serval	*L. serval*	2	0.01	PCR -
	—	—	Herpestidae	5	0.034	Egyptian mongoose	*H. ichneumon*	2	0.01	PCR -
	—	—	—	—	—	Banded mongoose	*Mungo mungo*	2	0.01	PCR -
	—	—	—	—	—	Meller's mongoose	*R. melleri*	1	0.01	PCR -
	—	—	Hyaenidae	27	0.184	Spotted Hyena	*C. crocuta*	25	0.17	PCR +
	—	—	—	—	—	Brown hyena	*Hyaena brunnea*	1	0.01	PCR +
	—	—	—	—	—	Aardwolf	*Proteles cristatus*	1	0.01	PCR -
	—	—	Viverridae	106	0.723	Common genet	*Genetta genetta*	5	0.03	PCR +
	—	—	—	—	—	Masked palm civet	*P. larvata*	101	0.69	PCR +

Caniformia	13,751	93.75	Canidae	11,037	75.251	—	—	—	—	—
	—	—	Domestic	5868	40.008	Dog	*C. lupus familiaris*	5868	40.01	PCR +
	—	—	Wild	5169	35.242	African wild dog	*L. pictus*	18	0.12	PCR +
	—	—	—	—	—	Arctic wolf	*C. lupus arctos*	1	0.01	PCR +
	—	—	—	—	—	Black-backed jackal	*Lupulella mesomelas*	92	0.63	PCR +
	—	—	—	—	—	Coyote	*C. latrans*	45	0.31	PCR +
	—	—	—	—	—	Golden jackal	*C. aureus*	87	0.59	PCR +
	—	—	—	—	—	European jackal	*C. aureus moreoticus*	24	0.16	PCR +
	—	—	—	—	—	Gray wolf	*C. lupus signatus*	223	1.52	PCR +
	—	—	—	—	—	Maned wolf	*Chrysocyon brachyurus*	3	0.02	PCR +
	—	—	—	—	—	Raccoon dog	*N. procyonoides*	152	1.04	PCR +
	—	—	—	—	—	Artic fox	*Vulpes lagopus*	5	0.03	PCR +
	—	—	—	—	—	Bat-eared fox	*O. megalotis*	6	0.04	PCR +
	—	—	—	—	—	Crab-eating fox	*C. thous*	23	0.16	PCR +
	—	—	—	—	—	Gray fox	*U. cinereoargenteus*	9	0.06	PCR +
	—	—	—	—	—	Hoary fox	*L. vetulus*	1	0.01	PCR +
	—	—	—	—	—	Pampas fox	*L. gymnocercus*	5	0.03	PCR -
	—	—	—	—	—	Island fox	*U. littoralis*	41	0.28	PCR +
	—	—	—	—	—	Red fox	*V. vulpes*	4434	30.23	PCR +
	—	—	Mephitidae	93	0.634	Striped skunk	*Mephitis mephitis*	93	0.63	PCR +
	—	—	Mustelidae	1425	9.716	American mink	*Neogale vison*	45	0.31	PCR +
	—	—	—	—	—	Badger	*M. meles*	471	3.21	PCR +
	—	—	—	—	—	American badger	*Taxidea taxus*	2	0.01	PCR +
	—	—	—	—	—	European otter	*L. lutra*	414	2.82	PCR +
	—	—	—	—	—	European mink	*Mustela lutreola*	1	0.01	PCR +
	—	—	—	—	—	European polecat	*M. putorius*	3	0.02	PCR +
	—	—	—	—	—	Free ranging ferret	*M. putorius furo*	225	1.53	PCR +
	—	—	—	—	—	Japanese weasel	*Mustela itatsi*	1	0.01	PCR +
	—	—	—	—	—	Least weasels	*M. nivalis*	10	0.07	PCR -
	—	—	—	—	—	Steppe polecat	*Mustela eversmanii*	64	0.44	PCR +
	—	—	—	—	—	Stoat	*M. erminea*	6	0.04	PCR -
	—	—	—	—	—	Siberian weasel	*Mustela sibirica*	1	0.01	PCR +
	—	—	—	—	—	Beech marten	*Martes foina*	105	0.72	PCR +
	—	—	—	—	—	Pine marten	*Martes martes*	51	0.35	PCR +
	—	—	—	—	—	Japanese marten	*Martes melampus*	1	0.01	PCR +
	—	—	—	—	—	Yellow-throated martens	*Martes flavigula*	1	0.01	PCR +
	—	—	—	—	—	Undetermined martens	*Martes sp*.	5	0.03	PCR +
	—	—	—	—	—	Sea otter	*Enhydra lutris*	18	0.12	PCR +
	—	—	—	—	—	Fisher	*Pekania pennantii*	1	0.01	PCR +
	—	—	Phocidae	33	0.225	Caspian Seal	*Pusa caspica*	14	0.10	PCR +
	—	—	—	—	—	Common seal	*Phoca vitulina*	17	0.12	PCR +
	—	—	—	—	—	Harp seal	*Pagophilus groenlandicus*	1	0.01	PCR +
	—	—	—	—	—	Hooded seal	*Cystophora cristata*	1	0.01	PCR +
	—	—	Procyonidae	1121	7.643	Raccoon	*P. lotor*	1061	7.23	PCR +
	—	—	—	—	—	White-nosed coati	*Nasua narica*	60	0.41	PCR +
	—	—	Ursidae	42	0.286	American black bear	*Ursus americanus*	14	0.10	PCR +
	—	—	—	—	—	Brown bear	*U. arctos marsicanus*	4	0.03	PCR +
	—	—	—	—	—	Giant panda	*A. melanoleuca*	2	0.01	PCR +
	—	—	—	—	—	Red panda	*Ailuropoda fulgens*	22	0.15	PCR +

Others	25	0.170	—	25	—	—	—	—	—	PCR +

Total	14,667	—	—	—	—	—	—	—	—	—

*Note:* Left rows: Values of the “Caniformia” and “Feliformia” suborders. Right rows: Values of every analyzed carnivore family. We have considered suborder, family, genus, and species as classificatory criteria. The different subspecies have been included in the species analyzed.

## Data Availability

The data that support the findings of this study are available from the corresponding author, N. Ortega (nortega@um.es), upon reasonable request.

## Supporting Information

Supporting Information 1 PRISMA checklist

Supporting Information 2 A list of the 160 articles included in this scoping review and spatial meta-analysis.

Supporting Information 3 A table with extra information about *Morbillivirus canis* (CDV) genotypes used in this scoping review. Other lineages/genotypes included in every category are specified: number of records in which every genotype appears; percentage of occurrence of every genotype out of the total of 303 records that classified the CDV genetic material into one of the main genotypes covered; the period of years in which every genotype was published; study citing every genotype for the first time; the oldest detected strain of every genotype: year of detection, species, country, study; most recent study citing every genotype; the most recent detected strain of every genotype: year of detection, species, country, study.
