# Supplementary material for: Geographical Distribution of Carnivore Hosts and Genotypes of Canine Distemper Virus (CDV) Worldwide: A Scoping Review and Spatial Meta-Analysis
Source: Transbound Emerg Dis. 2025 Mar 5;2025:6632068. doi: 10.1155/tbed/6632068 (PMC12016734; doi:10.1155/tbed/6632068)
Supplement: Supporting Information 2 — A list of the 160 articles included in this scoping review and spatial meta-analysis. [file 6632068.f2.doc]

**Table 1.** Articles included in the scoping review and meta-analysis (n = 160).

| **ID** | **Year** | **Title** | **Journal** | **Reference** |
| --- | --- | --- | --- | --- |
| 1 | 2024 | Molecular analysis of canine distemper virus H gene in the golden jackal (Canis aureus) population from Serbia | BMC Veterinary Research | Glišić et al., 2024 |
| 2 | 2024 | Study of canine distemper virus presence in Catalonia’s wild carnivores through h gene amplification and sequencing | Animals | Huang et al., 2024 |
| 3 | 2024 | Genome sequencing of canine distemper virus isolates from unvaccinated dogs in Mongolia | The Veterinary Journal | Munkhtsetseg et al., 2024 |
| 4 | 2024 | Canine distemper virus infection of vaccinal origin in a 14-week-old puppy | Journal of Veterinary Diagnostic Investigation | Rätsep & Ojkic, 2024 |
| 5 | 2023 | Detection of selected canine viruses in Nigerian free-ranging dogs traded for meat consumption | Animals | Ndiana et al., 2023 |
| 6 | 2023 | A comprehensive molecular survey of viral pathogens associated with canine gastroenteritis | Archives of Virology | Dema et al., 2023 |
| 7 | 2023 | Phylogenetic characterization of the canine distemper virus isolated from veterinary clinics in the Mekong Delta, Vietnam | Veterinary World | My Van et al., 2023 |
| 8 | 2023 | Canine distemper virus infection in the free-living wild canines, the red fox (*Vulpes vulpes)* and jackal (*Canis aureus moreoticus*), in Croatia | Pathogens | Prpić et al., 2023 |
| 9 | 2023 | Biomolecular analysis of canine distemper virus strains in two domestic ferrets (*Mustela putorius furo*) | Veterinary Sciences | Guercio et al., 2023 |
| 10 | 2023 | Exposure of wild Caspian seals (*Pusa caspica*) to parasites, bacterial and viral pathogens, evaluated via molecular and serological assays | Frontiers in Marine Science | Kydyrmanov et al., 2023 |
| 11 | 2023 | Nucleic acid amplification testing of urine samples from dogs with distemper-like disease | Revista Romana de Medicina Veterinaria | Șonea et al., 2023 |
| 12 | 2023 | First report of concurrent infection of canine kobuvirus and canine distemper virus in a diarrheic dog in India | Israel Journal of Veterinary Medicine | Agnihotri et al., 2023 |
| 13 | 2023 | Phylogenetic characterization of canine distemper virus from stray dogs in Kathmandu Valley | Virology Journal | Manandhar et al., 2023 |
| 14 | 2022 | Complete genomic sequencing of canine distemper virus with nanopore technology during an epizootic event | Scientific Reports | Lanszki et al., 2022a |
| 15 | 2022 | Pathological and molecular investigation of canine distemper virus: phylogenetic analysis of cocirculating genetic lineages based on h and f genes | Research Square | Karapınar et al., 2022 |
| 16 | 2022 | Canine distemper virus in wild carnivore populations from the Czech Republic (2012–2020): occurrence, geographical distribution, and phylogenetic analysis | Life | Kličková et al., 2022 |
| 17 | 2022 | Molecular and pathological screening of canine distemper virus in Asiatic lions, tigers, leopards, snow leopards, clouded leopards, leopard cats, jungle cats, civet cats, fishing cat, and jaguar of different states, India | Infection, Genetics and Evolution | Kadam et al., 2022 |
| 18 | 2022 | A novel and highly divergent canine distemper virus lineage causing distemper in ferrets in Australia | Virology | George et al., 2022 |
| 19 | 2022 | Canine distemper virus in autochtonous and imported dogs, Southern Italy (2014–2021) | Animals | Alfano et al., 2022 |
| 20 | 2022 | Detection and sequence analysis of canine morbillivirus in multiple species of the Mustelidae family | BMC Veterinary Research | Lanszki et al., 2022b |
| 21 | 2022 | Molecular detection and phylogenetic analysis of canine distemper virus in Marsican brown bear (*Ursus arctos marsicanus*) | Animals | Di Francesco et al., 2022 |
| 22 | 2022 | Neuropathologic and molecular aspects of a canine distemper epizootic in red foxes in Germany | Scientific Reports | Geiselhardt et al., 2022 |
| 23 | 2022 | The investigation of canine distemper virus in different diagnosis materials of dogs using molecular and pathological methods, Northeastern Turkey | Indian Journal of Animal Research | Yılmaz et al., 2022 |
| 24 | 2022 | Detection and genetic characterization of canine adenoviruses, circoviruses, and novel cycloviruses from wild carnivores in Italy | Frontiers in Veterinary Science | Ndiana et al., 2022 |
| 25 | 2022 | Retrospective detection and complete genomic sequencing of canine morbillivirus in Eurasian otter (*Lutra lutra*) using nanopore technology | Viruses | Lanszki et al., 2022c |
| 26 | 2022 | Molecular diagnosis with the corresponding clinical symptoms of canine distemper virus infection in Javan leopard (*Panthera pardus* ssp. *melas*) | Heliyon | Rahman et al., 2022 |
| 27 | 2022 | The first report and phylogenetic analysis of canine distemper virus in *Cerdocyon thous* from Colombia | Viruses | Echeverry-Bonilla et al., 2022 |
| 28 | 2022 | A new canine distemper virus lineage identified from red pandas in China | Transboundary and Emerging Diseases | Wang et al., 2022 |
| 29 | 2021 | Canine distemper virus in wildlife in South-Western Europe | Transboundary and Emerging Diseases | Oleaga et al., 2021 |
| 30 | 2021 | A canine distemper virus retrospective study conducted from 2011 to 2019 in Central Italy (Latium and Tuscany regions) | Viruses | Ricci et al., 2021 |
| 31 | 2021 | Prolonged infection of canine distemper virus in a mixed-breed dog | Veterinary Sciences | Lanszki et al., 2021 |
| 32 | 2021 | Canine distemper outbreaks in wild carnivores in Northern Italy | Viruses | Trogu et al., 2021 |
| 33 | 2021 | Natural distemper infection in stone martens (*Martes foina*): from infection to neutralizing antibodies | Research in Veterinary Science | Balboni et al., 2021 |
| 34 | 2021 | Genetic characterization of canine distemper virus from wild and domestic animal submissions to diagnostic facilities in Canada | Preventive Veterinary Medicine | Giacinti et al., 2021 |
| 35 | 2021 | Insight into an outbreak of canine distemper virus infection in masked  palm civets in China | Frontiers in Veterinary Science | Shi et al., 2021 |
| 36 | 2021 | Detection of systemic canine kobuvirus infection in peripheral tissues and the central nervous system of a fox infected with canine distemper virus | Microorganisms | Kaiser et al., 2021 |
| 37 | 2021 | Isolation and phylogenetic analysis of the canine distemper virus from a naturally infected dog in China | Indian Journal of Animal Research | Zhang et al., 2021 |
| 38 | 2021 | Randomly primed, strand-switching MinION-based sequencing for the detection and characterization of cultured RNA viruses | Journal of Veterinary Diagnostic Investigation | Young et al., 2021 |
| 39 | 2020 | Two waves of canine distemper virus showing different spatio-temporal dynamics in Alpine wildlife (2006–2018) | Infection Genetics and Evolution | Bianco et al., 2020 |
| 40 | 2020 | Canine distemper in neotropical procyonids: molecular evidence, humoral immune response and epidemiology | Virus Research | Rodriguez-Cabo-Mercado et al., 2020 |
| 41 | 2020 | Development and validation of a portable, point-of-care canine distemper virus qPCR test | PLoS One | Tomaszewicz Brown et al., 2020 |
| 42 | 2020 | Unveiling patterns of viral pathogen infection in free-ranging carnivores of northern Portugal using a complementary methodological approach | Comparative Immunology, Microbiology and Infectious Diseases | Rosa et al., 2020 |
| 43 | 2020 | First report of a canine morbillivirus infection in a giant anteater (*Myrmecophaga tridactyla*) in Brazil | Veterinary Medicine and Science | Belizario Granjeiro et al., 2020 |
| 44 | 2020 | H gene-based molecular characterization of field isolates of canine distemper virus from cases of canine gastroenteritis | Indian Journal of Animal Research | Kodi et al., 2020 |
| 45 | 2020 | Canine distemper virus in the sea otter (*Enhydra lutris*) population in Washington State, USA | Journal of Wildlife Diseases | Thomas et al., 2020 |
| 46 | 2020 | Virome of crab-eating (*Cerdocyon thous*) and pampas foxes (*Lycalopex gymnocercus*) from southern Brazil and Uruguay | Infection, Genetics and Evolution | Weber et al., 2020 |
| 47 | 2020 | Cross-species transmission and evolutionary dynamics of canine distemper virus during a spillover in African lions of Serengeti National Park | Molecular Ecology | Weckworth et al., 2020 |
| 48 | 2019 | Phylogenetic analysis of haemagglutinin gene deciphering a new genetically distinct lineage of canine distemper virus circulating among domestic dogs in India | Transboundary and Emerging Diseases | Bhatt et al., 2019 |
| 49 | 2019 | Phylogenetic evidence of the intercontinental circulation of a canine distemper virus lineage in the Americas | Scientific Reports | Duque-Valencia et al., 2019 |
| 50 | 2019 | Dual infection with an emergent strain of canine distemper virus and canine parvovirus in an Arctic wolf under managed care | Journal of Veterinary Diagnostic Investigation | Stilwell et al., 2019 |
| 51 | 2019 | Molecular analysis of the full-length F gene of Brazilian strains of canine distemper virus shows lineage co-circulation and variability between field and vaccine strains | Virus Research | Freitas et al., 2019 |
| 52 | 2019 | Diagnosis and characterization of canine distemper virus through sequencing by MinION nanopore technology | Scientific Reports | Peserico et al., 2019 |
| 53 | 2019 | Canine distemper virus in asiatic lions of Gujarat State, India | Emerging Infectious Diseases | Mourya et al., 2019 |
| 54 | 2018 | Evidence of two cocirculating canine distemper virus strains in mesocarnivores from Northern Colorado | Journal of Wildlife Diseases | Wostenberg et al., 2018 |
| 55 | 2018 | Phylogenetic analysis of canine distemper virus in South African wildlife | PLoS ONE | Loots et al., 2018 |
| 56 | 2018 | Evaluation of an incubation instrument-free reverse transcription recombinase polymerase amplification assay for rapid and point-of-need detection of canine distemper virus | Journal of Virological Methods | Wang, Wang, et al., 2018 |
| 57 | 2018 | A fast and simple one‑step duplex PCR assay for canine distemper virus (CDV) and canine coronavirus (CCoV) detection | Archives of Virology | Wang, Luo, et al., 2018 |
| 58 | 2018 | Morbillivirus-associated lipid pneumonia in Arctic foxes | Journal of Veterinary Diagnostic Investigation | Stimmelmayr et al., 2018 |
| 59 | 2018 | Molecular characterization of complete genome of a canine distemper virus associated with fatal infection in dogs in Gabon, Central Africa | Virus Research | Maganga et al., 2018 |
| 60 | 2018 | Detection and characterization of distemper virus in a mink (*Neovison vison*) in Turkey | Veterinaria Italiana | Oğuzoğlu, 2018 |
| 61 | 2018 | Genotypic evidence of infection by canine distemper virus in maned wolf from a zoological collection in Chile | International Journal of Scientific & Engineering Research | Abarca et al., 2018 |
| 62 | 2018 | Molecular surveillance of canine distemper virus in diarrhoetic puppies in Northeast China from May 2014 to April 2015 | Journal of Veterinary Medical Science | Li et al., 2018a |
| 63 | 2018 | Comparison of reverse-transcription real-time PCR and immunohistochemistry for the detection of canine distemper virus infection in raccoons in Ontario, Canada | Journal of Veterinary Diagnostic Investigation | Nemeth et al., 2018 |
| 64 | 2018 | Phylogenetic analysis of canine distemper viruses isolated from vaccinated dogs in Wuhan | Journal of Veterinary Medical Science | Li et al., 2018b |
| 65 | 2018 | Phylogenetic analysis of the wild-type strains of canine distemper virus circulating in the United States | Virology Journal | Anis et al., 2018 |
| 66 | 2017 | Canine distemper viral infection threatens the giant panda population in China | Oncotarget | Jin et al., 2017 |
| 67 | 2017 | Molecular characterization of canine distemper virus from Tamil Nadu, India | The Indian Journal of Animal Sciences | Ashmi et al., 2017 |
| 68 | 2017 | Detection of morbillivirus infection by RT-PCR RFLP analysis in cetaceans and carnivores. | Journal of Virological Methods | Verna et al., 2017 |
| 69 | 2017 | Canine distemper virus in the Serengeti ecosystem: molecular adaptation to different carnivore species | Molecular Ecology | Nikolin et al., 2017 |
| 70 | 2017 | The detection and differentiation of canine respiratory pathogens using oligonucleotide microarrays | Journal of Virological Methods | Wang et al., 2017 |
| 71 | 2017 | Pneumonia due to *Talaromyces marneffei* in a dog from Southern Brazil with concomitant canine distemper virus infection | Journal of Comparative Pathology | Headley et al., 2017 |
| 72 | 2016 | Lethal distemper in badgers (*Meles meles*) following epidemic in dogs and wolves | Infection, Genetics and Evolution | Di Sabatino et al., 2016 |
| 73 | 2016 | Characterization of a novel canine distemper virus causing disease in wildlife | Journal of Veterinary Diagnostic Investigation | Pope et al., 2016 |
| 74 | 2016 | RT-PCR and sequence analysis of the full-length fusion protein of Canine Distemper Virus from domestic dogs | Journal of Virological Methods | Romanutti et al., 2016 |
| 75 | 2016 | Phylogenetic analysis of canine distemper virus in South America clade 1 reveals unique molecular signatures of the local epidemic | Infection, Genetics and Evolution | Fischer et al., 2016 |
| 76 | 2015 | Patterns of exposure of Iberian wolves (*Canis lupus*) to canine viruses in human-dominated landscapes | EcoHealth | Millán et al., 2015 |
| 77 | 2016 | Coinfection with *Hepatozoon* sp. and Canine Distemper Virus in a Yellow-throated Marten (*Martes flavigula koreana*) in Korea | Journal of Wildlife Diseases | Park et al., 2016 |
| 78 | 2015 | Molecular detection and phylogenetic relationship of wild-type strains of canine distemper virus in symptomatic dogs from Uberlândia, Minas Gerais | Arquivo Brasileiro de Medicina Veterinária e Zootecnia | Headley et al., 2015 |
| 79 | 2015 | Isolation and sequence analysis of a canine distemper virus from a raccoon dog in Jilin Province, China | Virus Genes | Cheng et al., 2015 |
| 80 | 2015 | Detection and differentiation of wild-type and vaccine strains of canine distemper virus by a duplex reverse transcription polymerase chain reaction | Iranian Journal of Veterinary Research | Dong et al., 2015 |
| 81 | 2015 | Phylogenetic analysis of canine distemper virus in domestic dogs in Nanjing, China | Archives of Virology | Bi et al., 2015 |
| 82 | 2015 | Molecular cloning and sequence analysis of hemagglutinin gene of a novel strain canine distemper virus | International Journal of Applied Research in Veterinary Medicine | Wu et al., 2015 |
| 83 | 2015 | Clinical and molecular investigation of a canine distemper outbreak and vector-borne infections in a group of rescue dogs imported from Hungary to Switzerland | BMC Veterinary Research | Willi et al., 2015 |
| 84 | 2015 | Detection of Arctic and European cluster of canine distemper virus in north and center of Iran | Veterinary Research Forum | Namroodi et al., 2015 |
| 85 | 2015 | Sequencing of emerging canine distemper virus strain reveals new distinct genetic lineage in the United States associated with disease in wildlife and domestic canine populations | Virology Journal | Riley & Wilkes, 2015 |
| 86 | 2015 | Prevalence of canine infectious respiratory pathogens in asymptomatic dogs presented at US animal shelters | Journal of Small Animal Practice | Lavan & Knesl, 2015 |
| 87 | 2014 | Canine distemper outbreak in raccoons suggests pathogen interspecies transmission amongst alien and native carnivores in urban areas from Germany | Veterinary Microbiology | Rentería-Solís et al., 2014 |
| 88 | 2014 | Phylogenetic evidence of a new canine distemper virus lineage among domestic dogs in Colombia, South America | Veterinary Microbiology | Espinal et al., 2014 |
| 89 | 2014 | Arctic lineage-canine distemper virus as a cause of death in Apennine wolves (*Canis lupus*) in Italy | PLoS One | Di Sabatino et al., 2014 |
| 90 | 2014 | Molecular typing of canine distemper virus strains reveals the presence of a new genetic variant in South America | Virus Genes | Sarute et al., 2014 |
| 91 | 2014 | Genotyping of canine distemper virus strains circulating in Brazil from 2008 to 2012 | Virus Research | Budaszewski et al., 2014 |
| 92 | 2014 | Real-time reverse transcription polymerase chain reaction method for detection of Canine distemper virus modified live vaccine shedding for differentiation from infection with wild-type strains | Journal of Veterinary Diagnostic Investigation | Wilkes et al., 2014 |
| 93 | 2014 | Comparative survey of canine parvovirus, canine distemper virus and canine enteric coronavirus infection in free-ranging wolves of Central Italy and South-Eastern France | European Journal of Wildlife Research | Molnar et al., 2014 |
| 94 | 2014 | Occurrence of different canine distemper virus lineages in Italian dog | Veterinaria Italiana | Balboni et al., 2014 |
| 95 | 2013 | First report of clinical disease associated with canine distemper virus infection in a wild black bear (*Ursus americana*) | Journal of Wildlife Diseases | Cottrell et al., 2013 |
| 96 | 2013 | Genotypic lineages and restriction fragment length polymorphism of canine distemper virus isolates in Thailand | Veterinary Microbiology | Radtanakatikanon et al., 2013 |
| 97 | 2013 | Phylogenetic analysis of the haemagglutinin gene of canine distemper virus strains detected from giant panda and raccoon dogs in China | Virology Journal | Guo et al., 2013 |
| 98 | 2013 | Occurrence and geographical distribution of canine distemper virus infection in red foxes (*Vulpes vulpes*) of Saxony-Anhalt, Germany | Veterinary Microbiology | Denzin et al., 2013 |
| 99 | 2013 | Phylogenetic analyses of the hemagglutinin gene of wild-type strains of canine distemper virus in Southern Brazil | Genetics and Molecular Research | Negrão et al., 2013 |
| 100 | 2013 | Detection and differentiation of ﬁeld and vaccine strains of canine distemper virus using reverse transcription followed by nested real time PCR (RT-nqPCR) and RFLP analysis | Journal of Virological Methods | Fischer et al., 2013 |
| 101 | 2013 | Phylogenetic analysis of canine distemper viruses from red foxes, Greece | Veterinary Record | Billinis et al., 2013 |
| 102 | 2013 | Canine distemper virus: an emerging disease in wild endangered Amur tigers (*Panthera tigris altaica*) | mBio | Seimon et al., 2013 |
| 103 | 2012 | Detection by hemi-nested reverse transcription polymerase chain reaction and genetic characterization of wild type strains of canine distemper virus in suspected infected dogs | Journal of Veterinary Diagnostic Investigation | Di Francesco et al., 2012 |
| 104 | 2012 | Evidence of two co-circulating genetic lineages of canine distemper virus in South America | Virus Research | Panzera et al., 2012 |
| 105 | 2012 | Identification of new genovariants of canine distemper virus in dogs from the State of Mexico by analyzing the nucleocapsid gene | Archivos de medicina veterinaria | Gámiz-Mejía et al., 2012 |
| 106 | 2012 | Development of a combined canine distemper virus specific RT-PCR protocol for the differentiation of infected and vaccinated animals (DIVA) and genetic characterization of the hemagglutinin gene of seven Chinese strains demonstrated in dogs | Journal of Virological Methods | Yi et al., 2012 |
| 107 | 2012 | Epizootic canine distemper virus infection among wild mammals | Veterinary Microbiology | Kameo et al., 2012 |
| 108 | 2012 | Susceptibility of carnivore hosts to strains of canine distemper virus from distinct genetic lineages | Veterinary Microbiology | Nikolin et al., 2012 |
| 110 | 2012 | Emergence of canine distemper virus strains with modified molecular signature and enhanced neuronal tropism leading to high mortality in wild carnivores | Veterinary Pathology | Origgi et al., 2012 |
| 111 | 2012 | Detecção molecular e análise filogenética do gene H de amostras do vírus da cinomose canina em circulação no município de Campinas, São Paulo | Pesquisa Veterinaria Brasileira | Rosa et al., 2012 |
| 112 | 2011 | Emergence of canine distemper in Bavarian wildlife associated with a specific amino acid exchange in the haemagglutinin protein | The Veterinary Journal | Sekulin et al., 2011 |
| 113 | 2011 | A distinct CDV genotype causing a major epidemic in Alpine wildlife | Veterinary Microbiology | Monne et al., 2011 |
| 114 | 2011 | Atypical necrotizing encephalitis associated with systemic canine distemper virus infection in pups | Journal of Veterinary Science | Mendes Amude et al., 2011 |
| 115 | 2011 | Isolation and molecular characterization of canine distemper virus from India | Tropical Animal Health and Production | Pawar et al., 2011 |
| 116 | 2011 | Domestic dog origin of canine distemper virus in free-ranging wolves in Portugal as revealed by hemagglutinin gene characterization | Journal of Willife Disease | Müller et al., 2011 |
| 117 | 2011 | Pathogenesis and phylogenetic analyses of canine distemper virus strain ZJ7 isolate from domestic dogs in China | Virology Journal | Tan et al., 2011 |
| 118 | 2011 | Differentiation of canine distemper virus isolates in fur animals from various vaccine strains by reverse transcription-polymerase chain reaction-restriction fragment length polymorphism according to phylogenetic relations in china | Virology Journal | Wang et al., 2011 |
| 119 | 2010 | Importance of canine distemper virus (CDV) infection in free-ranging Iberian lynxes (*Lynx pardinus*) | Veterinary Microbiology | Meli et al., 2010 |
| 120 | 2010 | Prevalence of respiratory viruses isolated from dogs in Thailand during 2008-2009 | Asian Biomedicine | Posuwan et al., 2010 |
| 121 | 2010 | Natural infection with canine distemper virus in hand-feeding Rhesus monkeys in China | Veterinary Microbiology | Sun et al., 2010 |
| 122 | 2010 | Canine distemper epizootic among red foxes, Italy, 2009 | Emerging Infectious Diseases | Martella et al., 2010 |
| 123 | 2010 | Phylogenetic analysis of the haemagglutinin gene of current wild-type canine distemper viruses from South Africa: lineage Africa | Veterinary Microbiology | Woma et al., 2010 |
| 124 | 2010 | First identification of canine distemper virus in hoary fox (*Lycalopex vetulus*): pathologic aspects and virus phylogeny | Journal of Wildlife Diseases | Megid et al., 2010 |
| 125 | 2010 | Phylogenetic analysis of the haemagglutinin gene of canine distemper virus strains detected from breeding foxes, raccoon dogs and minks in China | Veterinary Microbiology | Zhao et al., 2010 |
| 126 | 2010 | Controversial results of the genetic analysis of a canine distemper vaccine strain | Veterinary Microbiology | Demeter et al., 2010 |
| 127 | 2009 | Broadly reactive pan-paramyxovirus reverse transcription polymerase chain reaction and sequence analysis for the detection of canine distemper virus in a case of canine meningoencephalitis of unknown etiology | Journal of Veterinary Diagnostic Investigation | Schatzberg et al., 2009 |
| 128 | 2009 | Canine distemper infection in crab-eating fox (*Cerdocyon thous*) from Argentina | Journal of Wildlife Diseases | Ferreyra et al., 2009 |
| 129 | 2009 | Canine distemper virus–associated encephalitis in free-living lynx (*Lynx canadensis*) and bobcats (*Lynx rufus*) of Eastern Canada | Journal of Wildlife Diseases | Daoust et al., 2009 |
| 130 | 2009 | A suspected canine distemper epidemic as the cause of a catastrophic decline in Santa Catalina Island foxes (*Urocyon littoralis catalinae*) | Journal of Wildlife Diseases | Timm et al., 2009 |
| 131 | 2009 | Canine distemper virus in a crab-eating fox (*Cerdocyon thous*) in Brazil: case report and phylogenetic analyses | Journal of Wildlife Diseases | Megid et al., 2009 |
| 132 | 2009 | Feline leukemia virus and other pathogens as important threats to the survival of the critically endangered Iberian lynx (*Lynx pardinus*) | PLoS One | Meli et al., 2009 |
| 133 | 2009 | Epidemiology, pathology, and genetic analysis of a canine distemper epidemic in Namibia | Journal of Wildlife Diseases | Gowtage-Sequeira et al., 2009 |
| 134 | 2008 | Canine distemper virus strains circulating among North American dogs | Clinical and Vaccine Immunology | Kapil et al., 2008 |
| 135 | 2008 | An observation of aberrant behavior in a raccoon (*Procyon lotor*) infected with canine distemper virus | Southeastern Naturalist | Richards et al., 2008 |
| 136 | 2008 | Identification of a genetic variant of canine distemper virus from clinical cases in two vaccinated dogs in Mexico | The Veterinary Journal | Simon-Martinez et al., 2008 |
| 137 | 2008 | Phylogenetic characterization of canine distemper virus isolates from naturally infected dogs and a marten in Korea | Veterinary Microbiology | An et al., 2008 |
| 138 | 2007 | Detection by RT-PCR and genetic characterization of canine distemper virus from vaccinated and non-vaccinated dogs in Argentina | Veterinary Microbiology | Gallo Calderon et al., 2007 |
| 139 | 2007 | Genetic diversity of Hungarian canine distemper virus strains | Veterinary Microbiology | Demeter et al., 2007 |
| 140 | 2007 | Genotyping canine distemper virus (CDV) by a hemi-nested multiplex PCR provides a rapid approach for investigation of CDV outbreaks | Veterinary Microbiology | Martella et al., 2007 |
| 141 | 2007 | Molecular analysis of the N gene of canine distemper virus in dogs in Brazil | Arquivo Brasileiro de Medicina Veterinária e Zootecnia | Castilho et al., 2007 |
| 142 | 2006 | Restriction pattern of a hemagglutinin gene amplified by RT-PCR from vaccine strains and wild-type canine distemper virus | Arquivo Brasileiro de Medicina Veterinária e Zootecnia | Negrão et al., 2006 |
| 143 | 2006 | Detection of canine distemper virus in dogs by real-time RT-PCR | Journal of Virological Methods | Elia et al., 2006 |
| 144 | 2006 | Heterogeneity within the hemagglutinin genes of canine distemper virus (CDV) strains detected in Italy | Veterinary Microbiology | Martella et al., 2006 |
| 145 | 2005 | Phylogenetic characterization of canine distemper viruses detected in naturally infected dogs in North America | Journal of Clinical Microbiology | Pardo et al., 2005 |
| 146 | 2005 | Pathogenesis and phylogenetic analyses of canine distemper virus strain 007Lm, a new isolate in dogs | Veterinary Microbiology | Lan et al., 2005 |
| 147 | 2004 | Effective primary isolation of wild-type Canine distemper virus in MDCK, MV1 Lu and Vero cells without nucleotide sequence changes within the entire haemagglutinin protein gene and in subgenomic sections of the fusion and phospho protein genes | Journal of Virological Methods | Lednicky et al., 2004 |
| 148 | 2004 | Determination and phylogenetic analysis of canine distemper virus in dogs with nervous symptoms in Turkey | Acta Veterinaria Hungarica | Özkul et al., 2004 |
| 149 | 2004 | Retrospective differentiation of canine distemper virus and phocine distemper virus in phocids | Journal of Wildlife Diseases | Stanton et al., 2004 |
| 150 | 2003 | A canine distemper outbreak in Alaska: diagnosis and strain characterization using sequence analysis | Journal of Veterinary Diagnostic Investigation | Maes et al., 2003 |
| 151 | 2003 | Canine distemper of vaccine origin in European mink, *Mustela lutreola -* a case report | Veterinary Microbiology | Ek-Kommonen et al., 2003 |
| 152 | 2002 | Distemper outbreak and its effect on African wild dog conservation | Emerging Infectious Diseases | Van De Bildt, 2002 |
| 153 | 2002 | Application of N-PCR for diagnosis of distemper in dogs and fur animals | Veterinary Microbiology | Rzezutka & Mizak, 2002 |
| 154 | 2002 | Detection and genetic characterization of canine distemper virus (CDV) from free-ranging red foxes in Italy | Molecular and Cellular Probes | Martella et al., 2002 |
| 155 | 2001 | Morbillivirus in common seals stranded on the coast of Belgium and northern France | Veterinary Record | Jauniaux et al., 2001 |
| 156 | 2000 | Epizootiological investigations of canine distemper virus in free-ranging carnivores from Germany | Veterinary Microbiology | Frölich et al., 2000 |
| 157 | 1999 | Detection of canine distemper virus nucleoprotein RNA by reverse transcription-PCR using serum, whole blood, and cerebrospinal fluid from dogs with distemper | Journal of Clinical Microbiology | Frisk et al., 1999 |
| 158 | 1999 | Genotypes of canine distemper virus determined by analysis of the hemagglutinin genes of recent isolates from dogs in Japan | Journal of Clinical Microbiology | Mochizuki et al., 1999 |
| 159 | 1996 | Canine distemper virus infection spotted hyaenas in Serengeti | Veterinary Microbiology | Haas, 1996 |
| 160 | 1995 | Detection of canine distemper virus nucleocapsid protein gene in canine peripheral blood mononuclear cells by RT-PCR | Journal of Veterinary Medical Science | Shin et al., 1995 |
| 161 | 1995 | Phylogenetic evidence of canine distemper virus in Serengeti’s lions | Vaccine | Harder et al., 1995 |

**REFERENCES**

Abarca, M. J., Hidalgo, E., Raggi, L., & Navarro, C. (2018). Genotypic evidence of infection by canine distemper virus in maned wolf from a zoological collection in Chile. *International Journal of Scientific & Engineering Research*, *9*(10).

Agnihotri, D., Maan, S., Batra, K., Kumar, A., Singh, Y, & Mor. S. K. (2023). First report of concurrent infection of canine kobuvirus and canine distemper virus in a diarrheic dog in India. *Israel Journal of Veterinary Medicine*, *78*(2).

Alfano, F., Lanave, G., Lucibelli, M. G., Miletti, G., D’Alessio, N., Gallo, A., Auriemma, C., Amoroso, M. G., Lucente, M. S., De Carlo, E., Martella, V., Decaro, N., & Fusco, G. (2022). Canine distemper virus in autochtonous and imported dogs, Southern Italy (2014–2021). *Animals*, *12*(20), 2852. https://doi.org/10.3390/ani12202852

An, D., Yoon, S., Park, J., No, I., & Park, B. (2008). Phylogenetic characterization of canine distemper virus isolates from naturally infected dogs and a marten in Korea. *Veterinary Microbiology, 132*(3–4), 389–395. https://doi.org/10.1016/j.vetmic.2008.05.025

Anis, E., Newell, T. K., Dyer, N., & Wilkes, R. P. (2018). Phylogenetic analysis of the wild-type strains of canine distemper virus circulating in the United States. *Virology Journal, 15*(1). https://doi.org/10.1186/s12985-018-1027-2

Ashmi, J. M., Thangavelu, A., Senthilkumar, T. M. A., & Manimaran, K. (2017). Molecular characterization of canine distemper virus from Tamil Nadu, India. *Indian Journal of Animal Sciences*, *87*(9), 1062-1067.

Balboni, A., Dandola, G. D. L., Scagliarini, A., Prosperi, S., & Battilani, M. (2014). Occurrence of different canine distemper virus lineages in Italian dogs. *Veterinaria Italiana*, *50*(3), 227-231.

Balboni, A., Savini, F., Scagliarini, A., Berti, E., Naldi, M., Urbani, L., Fontana, M. C., Carra, E., Gibelli, L. R. M., Gobbo, F., Bologna, E., Zambelli, D., Ceccherelli, R., & Battilani, M. (2021). Natural distemper infection in stone martens (*Martes foina*): from infection to neutralizing antibodies. *Research in Veterinary Science*, *138*, 196-200. https://doi.org/10.1016/j.rvsc.2021.06.015

Belizario Granjeiro, M. D., Kavasaki, M. L., Morgado, T. O., Dandolini Pavelegini, L. A., de Barros, M. A., Fontana, C., Bianchini, M. de A., Souza, A. de O., Goncalves Lima Oliveira Santos, A. R., Lunardi, M., Colodel, E. M., de Aguiar, D. M., & Mendonca, A. J. (2020). First report of a canine morbillivirus infection in a giant anteater (*Myrmecophaga tridactyla*) in Brazil. *Veterinary Medicine and Science*, *6*(3), 606-611. https://doi.org/10.1002/vms3.246

Bhatt, M., Rajak, K. K., Chakravarti, S., Yadav, A. K., Kumar, A., Gupta, V., Chander, V., Mathesh, K., Chandramohan, S., Sharma, A. K., Mahendran, K., Sankar, M., Muthuchelvan, D., Gandham, R. K., Baig, M., Singh, R. P., & Singh, R. K. (2019). Phylogenetic analysis of haemagglutinin gene deciphering a new genetically distinct lineage of canine distemper virus circulating among domestic dogs in India. *Transboundary and Emerging Diseases, 66*(3), 1252–1267. https://doi.org/10.1111/tbed.13142

Bi, Z., Wang, Y., Wang, X., & Xia, X. (2015). Phylogenetic analysis of canine distemper virus in domestic dogs in Nanjing, China. *Archives of Virology*, *160*(2), 523-527. https://doi.org/10.1007/s00705-014-2293-y

Bianco, A., Zecchin, B., Fusaro, A., Schivo, A., Ormelli, S., Bregoli, M., Citterio, C. V., Obber, F., Dellamaria, D., Trevisiol, K., Lorenzetto, M., De Benedictis, P., & Monne, I. (2020). Two waves of canine distemper virus showing different spatio-temporal dynamics in Alpine wildlife (2006–2018). *Infection Genetics and Evolution, 84*, 104359. https://doi.org/10.1016/j.meegid.2020.104359

Billinis, C., Athanasiou, L. V., Valiakos, G., Mamuris, Z., Birtsas, P., & Spyrou, V. (2013). Phylogenetic analysis of canine distemper viruses from red foxes, Greece. *Veterinary Record*, *173*(8), 194-194. https://doi.org/10.1136/vr.101618

Budaszewski, R. da F., Pinto, L. D., Weber, M. N., Caldart, E. T., Beduschi Travassos Alves, C. D., Martella, V., Ikuta, N., Lunge, V. R., & Canal, C. W. (2014). Genotyping of canine distemper virus strains circulating in Brazil from 2008 to 2012. *Virus Research*, *180*, 76-83. https://doi.org/10.1016/j.virusres.2013.12.024

Castilho, J. G., Brandão, P. E., Carnieli Jr, P., Oliveira, R. N., Macedo, C. I., Peixoto, Z. M. P., Carrieri, M. L., & Kotait, I. (2007). Molecular analysis of the N gene of canine distemper virus in dogs in Brazil. *Arquivo Brasileiro de Medicina Veterinária e Zootecnia*, *59*(3), 654-659. https://doi.org/10.1590/S0102-09352007000300016

Cheng, Y., Wang, J., Zhang, M., Zhao, J., Shao, X., Ma, Z., Zhao, H., Lin, P., & Wu, H. (2015). Isolation and sequence analysis of a canine distemper virus from a raccoon dog in Jilin Province, China. *Virus Genes*, *51*(2), 298-301. https://doi.org/10.1007/s11262-015-1236-3

Cottrell, W. O., Keel, M. K., Brooks, J. W., Mead, D. G., & Phillips, J. E. (2013). First report of clinical disease associated with canine distemper virus infection in a wild black bear (*Ursus americana*). *Journal of Wildlife Diseases*, *49*(4), 1024-1027. https://doi.org/10.7589/2013-02-027

Daoust, P.-Y., McBurney, S. R., Godson, D. L., Van De Bildt, M. W. G., & Osterhaus, A. D. M. E. (2009). Canine distemper virus–associated encephalitis in free-living lynx *(Lynx canadensis*) and bobcats (lynx rufus) of Eastern Canada. *Journal of Wildlife Diseases*, *45*(3), 611-624. https://doi.org/10.7589/0090-3558-45.3.611

Dema, A., Tallapally, M. R., Ganji, V. K., Buddala, B., Kodi, H., Ramidi, A., Yella, N. R., & Putty, K. (2023). A comprehensive molecular survey of viral pathogens associated with canine gastroenteritis. *Archives of Virology*, *168*(2), 36. https://doi.org/10.1007/s00705-022-05674-6

Demeter, Z., Lakatos, B., Palade, E. A., Kozmac, T., Forgach, P., & Rusvai, M. (2007). Genetic diversity of Hungarian canine distemper virus strains. *Veterinary Microbiology*, *122*(3-4), 258-269. https://doi.org/10.1016/j.vetmic.2007.02.001

Demeter, Z., Palade, E. A., Hornyák, Á., & Rusvai, M. (2010). Controversial results of the genetic analysis of a canine distemper vaccine strain. *Veterinary Microbiology, 142*(3–4), 420–426. https://doi.org/10.1016/j.vetmic.2009.10.017

Denzin, N., Herwig, V., & van der Grinten, E. (2013). Occurrence and geographical distribution of canine distemper virus infection in red foxes (*Vulpes vulpes*) of Saxony-Anhalt, Germany. *Veterinary Microbiology*, *162*(1), 214-218. https://doi.org/10.1016/j.vetmic.2012.08.031

Di Francesco, C. E., Di Francesco, D., Di Martino, B., Speranza, R., Santori, D., Boari, A., & Marsilio, F. (2012). Detection by hemi-nested reverse transcription polymerase chain reaction and genetic characterization of wild type strains of canine distemper virus in suspected infected dogs. *Journal of Veterinary Diagnostic Investigation*, *24*(1), 107-115. https://doi.org/10.1177/1040638711425700

Di Francesco, C. E., Smoglica, C., Di Pirro, V., Cafini, F., Gentile, L., & Marsilio, F. (2022). Molecular detection and phylogenetic analysis of canine distemper virus in Marsican brown bear (*Ursus arctos marsicanus*). *Animals*, *12*(14), 1826. https://doi.org/10.3390/ani12141826

Di Sabatino, D., Di Francesco, G., Zaccaria, G., Malatesta, D., Brugnola, L., Marcacci, M., Portanti, O., De Massis, F., Savini, G., Teodori, L., Ruggieri, E., Mangone, I., Badagliacca, P., & Lorusso, A. (2016). Lethal distemper in badgers (*Meles meles*) following epidemic in dogs and wolves. *Infection Genetics and Evolution*, *46*, 130-137. https://doi.org/10.1016/j.meegid.2016.10.020

Di Sabatino, D., Lorusso, A., Di Francesco, C. E., Gentile, L., Di Pirro, V., Bellacicco, A. L., Giovannini, A., Di Francesco, G., Marruchella, G., Marsilio, F., & Savini, G. (2014). Arctic lineage-canine distemper virus as a cause of death in Apennine wolves (*Canis lupus*) in Italy. *PloS One*, *9*(1), e82356. https://doi.org/10.1371/journal.pone.0082356

Dong, X. Y., Li, W. H., Zhu, J. L., Liu, W. J., Zhao, M. Q., Luo, Y. W., & Chen, J. D. (2015). Detection and differentiation of wild-type and vaccine strains of canine distemper virus by a duplex reverse transcription. Polymerase chain reaction. *Iranian Journal of Veterinary Research*, *16*(2), 172-175.

Duque-Valencia, J., Forero-Munoz, N. R., Diaz, F. J., Martins, E., Barato, P., & Ruiz-Saenz, J. (2019). Phylogenetic evidence of the intercontinental circulation of a canine distemper virus lineage in the Americas. *Scientific Reports*, *9*, 15747. https://doi.org/10.1038/s41598-019-52345-9

Echeverry-Bonilla, D. F., Buriticá-Gaviria, E. F., Orjuela-Acosta, D., Chinchilla-Cardenas, D. J., & Ruiz-Saenz, J. (2022). The first report and phylogenetic analysis of canine distemper virus in *Cerdocyon thous* from Colombia. *Viruses, 14*(9), 1947. https://doi.org/10.3390/v14091947

Ek-Kommonen, C., Rudbäck, E., Anttila, M., Aho, M., & Huovilainen, A. (2003). Canine distemper of vaccine origin in European mink, *Mustela lutreola*—A case report. *Veterinary Microbiology*, *92*(3), 289-293. https://doi.org/10.1016/s0378-1135(02)00361-9

Elia, G., Decaro, N., Martella, V., Cirone, F., Lucente, M. S., Lorusso, E., Di Trani, L., & Buonavoglia, C. (2006). Detection of canine distemper virus in dogs by real-time RT-PCR. *Journal of Virological Methods*, *136*(1-2), 171-176. https://doi.org/10.1016/j.jviromet.2006.05.004

Espinal, M. A., Díaz, F. J., & Ruiz-Saenz, J. (2014). Phylogenetic evidence of a new canine distemper virus lineage among domestic dogs in Colombia, South America. *Veterinary Microbiology*, *172*(1-2), 168-176. https://doi.org/10.1016/j.vetmic.2014.05.019

Ferreyra, H., Calderón, M. G., Marticorena, D. N., Marull, C., & Leonardo, B. C. (2009). Canine distemper infection in crab-eating fox (*Cerdocyon thous*) from Argentina. *Journal of Wildlife Diseases*, *45*(4), 1158-1162. https://doi.org/10.7589/0090-3558-45.4.1158

Fischer, C. D. B., Ikuta, N., Canal, C. W., Makiejczuk, A., Allgayer, M. D. C., Cardoso, C. H., Lehmann, F. K., Fonseca, A. S. K., & Lunge, V. R. (2013). Detection and differentiation of field and vaccine strains of canine distemper virus using reverse transcription followed by nested real time PCR (RT-nqPCR) and RFLP analysis. *Journal of Virological Methods*, *194*(1-2), 39-45. https://doi.org/10.1016/j.jviromet.2013.08.002

Fischer, C. D., Gräf, T., Ikuta, N., Lehmann, F. K., Passos, D. T., Makiejczuk, A., Silveira, M. A., Fonseca, A. S., Canal, C. W., & Lunge, V. R. (2016). Phylogenetic analysis of canine distemper virus in South America clade 1 reveals unique molecular signatures of the local epidemic. *Infection Genetics and Evolution, 41*, 135–141. https://doi.org/10.1016/j.meegid.2016.03.029

Freitas, L. A., Leme, R. A., Saporiti, V., Alfieri, A. A., & Alfieri, A. F. (2019). Molecular analysis of the full-length F gene of Brazilian strains of canine distemper virus shows lineage co-circulation and variability between field and vaccine strains. *Virus Research*, *264*, 8-15. https://doi.org/10.1016/j.virusres.2019.02.009

Frisk, A. L., König, M., Moritz, A., & Baumgärtner, W. (1999). Detection of canine distemper virus nucleoprotein RNA by reverse transcription-PCR using serum, whole blood, and cerebrospinal fluid from dogs with distemper. *Journal of Clinical Microbiology*, *37*(11), 3634-3643. https://doi.org/10.1128/JCM.37.11.3634-3643.1999

Frölich, K., Czupalla, O., Haas, L., Hentschke, J., Dedek, J., & Fickel, J. (2000). Epizootiological investigations of canine distemper virus in free-ranging carnivores from Germany. *Veterinary Microbiology*, *74*(4), 283-292. https://doi.org/10.1016/s0378-1135(00)00192-9

Gallo Calderon, M., Remorini, P., Periolo, O., Iglesias, M., Mattion, N., & La Torre, J. (2007). Detection by RT-PCR and genetic characterization of canine distemper virus from vaccinated and non-vaccinated dogs in Argentina. *Veterinary Microbiology*, *125*(3-4), 341-349. https://doi.org/10.1016/j.vetmic.2007.05.020

Gámiz-Mejía, C., Simón-Martínez, J., & Fajardo-Muñoz, R. (2012). Identification of new genovariants of canine distemper virus in dogs from the State of Mexico by analyzing the nucleocapsid gene. *Archivos de Medicina Veterinaria*, *44*(1), 53-58. https://doi.org/10.4067/S0301-732X2012000100008

Geiselhardt, F., Peters, M., Kleinschmidt, S., Chludzinski, E., Stoff, M., Ludlow, M., & Beineke, A. (2022). Neuropathologic and molecular aspects of a canine distemper epizootic in red foxes in Germany. *Scientific Reports*, *12*(1), 14691. https://doi.org/10.1038/s41598-022-19023-9

George, A. M., Wille, M., Wang, J., Anderson, K., Cohen, S., Moselen, J., Lee, L. Y. Y., Suen, W. W., Bingham, J., Dalziel, A. E., Whitney, P., Stannard, H., Hurt, A. C., Williams, D. T., Deng, Y.-M., & Barr, I. G. (2022). A novel and highly divergent canine distemper virus lineage causing distemper in ferrets in Australia. *Virology*, *576*, 117-126. https://doi.org/10.1016/j.virol.2022.09.001

Giacinti, J. A., Pearl, D. L., Ojkic, D., Campbell, G. D., & Jardine, C. M. (2021). Genetic characterization of canine distemper virus from wild and domestic animal submissions to diagnostic facilities in Canada. *Preventive Veterinary Medicine, 198*, 105535. https://doi.org/10.1016/j.prevetmed.2021.105535

Glišić, D., Kuručki, M., Ćirović, D., Šolaja, S., Mirčeta, J., & Milićević, V. (2024). Molecular analysis of canine distemper virus H gene in the golden jackal (*Canis aureus*) population from Serbia. *BMC Veterinary Research, 20*(1). https://doi.org/10.1186/s12917-024-04284-5

Gowtage-Sequeira, S., Banyard, A. C., Barrett, T., Buczkowski, H., Funk, S. M., & Cleaveland, S. (2009). Epidemiology, pathology, and genetic analysis of a canine distemper epidemic in Namibia*. Journal of Wildlife Diseases, 45*(4), 1008–1020. https://doi.org/10.7589/0090-3558-45.4.1008

Guercio, A., Mira, F., Di Bella, S., Gucciardi, F., Lastra, A., Purpari, G., Castronovo, C., Pennisi, M., Di Marco Lo Presti, V., Rizzo, M., & Giudice, E. (2023). Biomolecular analysis of canine distemper virus strains in two domestic ferrets (*Mustela putorius furo*). *Veterinary Sciences*, *10*(6). https://doi.org/10.3390/vetsci10060375

Guo, L., Yang, S., Wang, C., Hou, R., Chen, S., Yang, X., Liu, J., Pan, H., Hao, Z., Zhang, M., Cao, S., & Yan, Q. (2013). Phylogenetic analysis of the haemagglutinin gene of canine distemper virus strains detected from giant panda and raccoon dogs in China. *Virology Journal*, *10*, 109. https://doi.org/10.1186/1743-422X-10-109

Haas, L. (1996). Canine distemper virus infection in Serengeti spotted hyaenas. *Veterinary Microbiology*, *49*(1-2), 147-152. https://doi.org/10.1016/0378-1135(95)00180-8

Harder, T. C., Kenter, M., Appel, M. J. G., Roelke-Parker, M. E., Barrett, T., & Osterhaus, A. D. M. E. (1995). Phylogenetic evidence of canine distemper virus in Serengeti’s lions. *Vaccine*, *13*(6), 521-523. https://doi.org/10.1016/0264-410X(95)00024-U

Headley, S. A., Pretto-Giordano, L. G., Lima, S. C., Suhett, W. G., Pereira, A. H. T., Freitas, L. A., Suphoronski, S. A., Oliveira, T. E. S., Alfieri, A. F., Pereira, E. C., Vitas-Boas, L. A., & Alfieri, A. A. (2017). Pneumonia due to *Talaromyces marneffei* in a dog from southern brazil with concomitant canine distemper virus infection. *Journal of Comparative Pathology*, *157*(1), 61-66. https://doi.org/10.1016/j.jcpa.2017.06.001

Headley, S. A., Santos, T. R., Bodnar, L., Saut, J. P. E., Silva, A. P., Alfieri, A. F., Medeiros, A. A., Soares, N. P., & Alfieri, A. A. (2015). Molecular detection and phylogenetic relationship of wild-type strains of canine distemper virus in symptomatic dogs from Uberlândia, Minas Gerais. *Arquivo Brasileiro de Medicina Veterinária e Zootecnia*, *67*(6), 1510-1518. https://doi.org/10.1590/1678-4162-7052

Huang, J., Cortey, M., Darwich, L., Griffin, J., Obón, E., Molina, R., & Martín, M. (2024). study of canine distemper virus presence in Catalonia’s wild carnivores through H gene amplification and sequencing. *Animals*, *14*(3), 436. https://doi.org/10.3390/ani14030436

Jauniaux, T., Boseret, G., Coignoul, F., Desmecht, M., Haelters, J., Manteca, C., Tavernier, J., & Van Gompel, J. (2001). Morbillivirus in common seals stranded on the coasts of Belgium and Northern France during summer 1998. *Veterinary Record*, *148*(19), 587-591. https://doi.org/10.1136/vr.148.19.587

Jin, Y., Zhang, X., Ma, Y., Qiao, Y., Liu, X., Zhao, K., Zhang, C., Lin, D., Fu, X., Xu, X., Wang, Y., & Wang, H. (2017). Canine distemper viral infection threatens the giant panda population in China. *Oncotarget*, *8*(69), 113910-113919. https://doi.org/10.18632/oncotarget.23042

Kadam, R. G., Karikalan, M., Siddappa, C. M., Mahendran, K., Srivastava, G., Rajak, K. K., Bhardwaj, Y., Varshney, R., War, Z. A., Singh, R., Ghosh, M., Beena, V., Pawde, A. M., Singh, K. P., & Sharma, A. K. (2022). Molecular and pathological screening of canine distemper virus in Asiatic lions, tigers, leopards, snow leopards, clouded leopards, leopard cats, jungle cats, civet cats, fishing cat, and jaguar of different states, India. *Infection, Genetics and Evolution*, *98*, 105211. https://doi.org/10.1016/j.meegid.2022.105211

Kaiser, F. K., Van Dyck, L., Jo, W. K., Schreiner, T., Pfankuche, V. M., Wohlsein, P., Baumann, I., Peters, M., Baumgärtner, W., Osterhaus, A. D. M. E., & Ludlow, M. (2021). Detection of systemic canine kobuvirus infection in peripheral tissues and the central nervous system of a fox infected with canine distemper virus. *Microorganisms*, *9*(12), 2521. https://doi.org/10.3390/microorganisms9122521

Kameo, Y., Nagao, Y., Nishio, Y., Shimoda, H., Nakano, H., Suzuki, K., Une, Y., Sato, H., Shimojima, M., & Maeda, K. (2011). Epizootic canine distemper virus infection among wild mammals. *Veterinary Microbiology, 154*(3–4), 222–229. https://doi.org/10.1016/j.vetmic.2011.07.006

Kapil, S., Allison, R. W., Johnston, L., Murray, B. L., Holland, S., Meinkoth, J., & Johnson, B. (2008). Canine distemper virus strains circulating among North American dogs. *Clinical and Vaccine Immunology*, *15*(4), 707-712. https://doi.org/10.1128/CVI.00005-08

Karapınar, Z., İLhan, F., Usta, M., & Timurkan, M. Ö. (2022). Pathological and Molecular Investigation of Canine Distemper Virus: Phylogenetic analysis of co-circulating genetic lineages based on H and F genes, PREPRINT (Version 1). *Research Square*, 17 April 2022. https://doi.org/10.21203/rs.3.rs-1537464/v1

Kličková, E., Černíková, L., Dumondin, A., Bártová, E., Budíková, M., & Sedlák, K. (2022). Canine distemper virus in wild carnivore populations from the Czech Republic (2012-2020): occurrence, geographical distribution, and phylogenetic analysis. *Life*, *12*(2). https://doi.org/10.3390/life12020289

Kodi, H., Putty, K., Ganji, V. K., Bhagyalakshmi, B., Reddy, Y. N., Satish, K., & Prakash, M. G. (2020). H gene-based molecular characterization of field isolates of canine distemper virus from cases of canine gastroenteritis. *Indian Journal of Animal Research, 55*(5). https://doi.org/10.18805/ijar.B-3989

Kydyrmanov, A., Karamendin, K., Kassymbekov, Y., Kumar, M., Mazkirat, S., Suleimenova, S., Baimukanov, M., Carr, I. M., & Goodman, S. J. (2023). Exposure of wild Caspian seals (*Pusa caspica*) to parasites, bacterial and viral pathogens, evaluated via molecular and serological assays. *Frontiers in Marine Science*, *10*, 1087997. https://doi.org/10.3389/fmars.2023.1087997

Lan, N., Yamaguchi, R., Furuya, Y., Inomata, A., Ngamkala, S., Naganobu, K., Kai, K., Mochizuki, M., Kobayashi, Y., & Uchida, K. (2005). Pathogenesis and phylogenetic analyses of canine distemper virus strain 007Lm, a new isolate in dogs. *Veterinary Microbiology*, *110*(3-4), 197-207. https://doi.org/10.1016/j.vetmic.2005.07.016

Lanszki, Z., Lanszki, J., Tóth, G. E., Cserkész, T., Csorba, G., Görföl, T., Csathó, A. I., Jakab, F., & Kemenesi, G. (2022b). Detection and sequence analysis of canine morbillivirus in multiple species of the Mustelidae family. *BMC Veterinary Research*, *18*(1), 450. https://doi.org/10.1186/s12917-022-03551-7

Lanszki, Z., Lanszki, J., Tóth, G. E., Zeghbib, S., Jakab, F., & Kemenesi, G. (2022c). Retrospective detection and complete genomic sequencing of canine morbillivirus in Eurasian otter (*Lutra lutra*) using nanopore technology. *Viruses*, *14*(7). https://doi.org/10.3390/v14071433

Lanszki, Z., Tóth, G. E., Schütz, É., Zeghbib, S., Rusvai, M., Jakab, F., & Kemenesi, G. (2022a). Complete genomic sequencing of canine distemper virus with nanopore technology during an epizootic event. *Scientific Reports 12*(1). https://doi.org/10.1038/s41598-022-08183-3

Lanszki, Z., Zana, B., Zeghbib, S., Jakab, F., Szabó, N., & Kemenesi, G. (2021). Prolonged infection of canine distemper virus in a mixed-breed dog. *Veterinary Sciences*, *8*(4). https://doi.org/10.3390/vetsci8040061

Lavan, R., & Knesl, O. (2015). Prevalence of canine infectious respiratory pathogens in asymptomatic dogs presented at US animal shelters. *Journal of Small Animal Practice*, *56*(9), 572-576. https://doi.org/10.1111/jsap.12389

Lednicky, J. A., Meehan, T. P., Kinsel, M. J., Dubach, J., Hungerford, L. L., Sarich, N. A., Witecki, K. E., Braid, M. D., Pedrak, C., & Houde, C. M. (2004). Effective primary isolation of wild-type canine distemper virus in MDCK, MV1 Lu and Vero cells without nucleotide sequence changes within the entire haemagglutinin protein gene and in subgenomic sections of the fusion and phospho protein genes. *Journal of Virological Methods*, *118*(2), 147-157. https://doi.org/10.1016/j.jviromet.2004.02.004

Li, W., Cai, C., Xue, M., Xu, G., Wang, X., Zhang, A., & Han, L. (2018b). Phylogenetic analysis of canine distemper virusesisolated from vaccinated dogs in Wuhan. *Journal of Veterinary Medical Science*, *80*(11), 1688–1690. https://doi.org/10.1292/jvms.18-0116

Li, C., Guo, D., Wu, R., Kong, F., Zhai, J., Yuan, D., & Sun, D. (2018a). Molecular surveillance of canine distemper virus in diarrhoetic puppies in northeast China from May 2014 to April 2015. *Journal of Veterinary Medical Science*, *80*(6), 1029-1033. https://doi.org/10.1292/jvms.17-0559

Loots, A. K., Mokgokong, P. S., Mitchell, E., Venter, E. H., Kotze, A., & Dalton, D. L. (2018). Phylogenetic analysis of canine distemper virus in South African wildlife. *PLoS ONE, 13*(7), e0199993. https://doi.org/10.1371/journal.pone.0199993

Maes, R. K., Wise, A. G., Fitzgerald, S. D., Rarnudo, A., Kline, J., Vilnis, A., & Benson, C. (2003). A canine distemper outbreak in Alaska: diagnosis and strain characterization using sequence analysis. *Journal of Veterinary Diagnostic Investigation*, *15*(3), 213-220. https://doi.org/10.1177/104063870301500302

Maganga, G. D., Labouba, I., Ngoubangoye, B., Nkili-Meyong, A. A., Ondo, D. O., Leroy, E. M., & Berthet, N. (2018). Molecular characterization of complete genome of a canine distemper virus associated with fatal infection in dogs in Gabon, Central Africa. *Virus Research*, *247*, 21-25. https://doi.org/10.1016/j.virusres.2018.01.012

Manandhar, P., Napit, R., Pradhan, S. M., Rajbhandari, P. G., Moravek, J. A., Joshi, P. R., Shrestha, R. D., & Karmacharya, D. (2023). Phylogenetic characterization of canine distemper virus from stray dogs in Kathmandu Valley. *Virology Journal*, *20*(1), 117. https://doi.org/10.1186/s12985-023-02071-6

Martella, V., Bianchi, A., Bertoletti, I., Pedrotti, L., Gugiatti, A., Catella, A., Cordioli, P., Lucente, M. S., Elia, G., & Buonavoglia, C. (2010). Canine distemper epizootic among red foxes, Italy, 2009. *Emerging Infectious Diseases*, *16*(12), 2007-2009. https://doi.org/10.3201/eid1612.100579

Martella, V., Cirone, F., Elia, G., Lorusso, E., Decaro, N., Campolo, M., Desario, C., Lucente, M. S., Bellacicco, A. L., Blixenkrone-Møller, M., Carmichael, L. E., & Buonavoglia, C. (2006). Heterogeneity within the hemagglutinin genes of canine distemper virus (CDV) strains detected in Italy. *Veterinary Microbiology*, *116*(4), 301-309. https://doi.org/10.1016/j.vetmic.2006.04.019

Martella, V., Elia, G., Lucente, M. S., Decaro, N., Lorusso, E., Banyai, K., Blixenkrone-Møller, M., Lan, N. T., Yamaguchi, R., Cirone, F., Carmichael, L. E., & Buonavoglia, C. (2007). Genotyping canine distemper virus (CDV) by a hemi-nested multiplex PCR provides a rapid approach for investigation of CDV outbreaks. *Veterinary Microbiology*, *122*(1-2), 32-42. https://doi.org/10.1016/j.vetmic.2007.01.005

Martella, V., Pratelli, A., Cirone, F., Zizzo, N., Decaro, N., Tinelli, A., Foti, M., & Buonavoglia, C. (2002). Detection and genetic characterization of canine distemper virus (CDV) from free-ranging red foxes in Italy. *Molecular and Cellular Probes*, *16*(1), 77-83. https://doi.org/10.1006/mcpr.2001.0387

Megid, J., De Souza, V. A. F., Teixeira, C. R., Cortez, A., Amorin, R. L., Heinemman, M. B., Cagnini, D. Q., & Richtzenhain, L. J. (2009). Canine distemper virus in a crab-eating fox (*Cerdocyon thous*) in Brazil: case report and phylogenetic analyses. *Journal of Wildlife Diseases*, *45*(2), 527-530. https://doi.org/10.7589/0090-3558-45.2.527

Megid, J., Teixeira, C. R., Amorin, R. L., Cortez, A., Heinemann, M. B., Azevedo de Paula Antunes, J. M., da Costa, L. F., Fornazari, F., Bosso Cipriano, J. R., Cremasco, A., & Richtzenhain, L. J. (2010). First identification of canine distemper virus in hoary fox (*Lycalopex vetulus*): pathologic aspects and virus phylogeny. *Journal of Wildlife Diseases*, *46*(1), 303-305. https://doi.org/10.7589/0090-3558-46.1.303

Meli, M. L., Cattori, V., Martínez, F., López, G., Vargas, A., Simón, M. A., Zorrilla, I., Muñoz, A., Palomares, F., López-Bao, J. V., Pastor, J., Tandon, R., Willi, B., Hofmann-Lehmann, R., & Lutz, H. (2009). Feline leukemia virus and other pathogens as important threats to the survival of the critically endangered Iberian lynx (*Lynx pardinus*). *PLoS One*, *4*(3), e4744. https://doi.org/10.1371/journal.pone.0004744

Meli, M. L., Simmler, P., Cattori, V., Martínez, F., Vargas, A., Palomares, F., López-Bao, J. V., Simón, M. A., López, G., León-Vizcaino, L., Hofmann-Lehmann, R., & Lutz, H. (2010). Importance of canine distemper virus (CDV) infection in free-ranging Iberian lynxes (*Lynx pardinus*). *Veterinary Microbiology*, *146*(1-2), 132-137. https://doi.org/10.1016/j.vetmic.2010.04.024

Mendes Amude, A., Arlington Headley, S., Alcindo Alfieri, A., Esteves Beloni, S. N., & Fernandes Alfieri, A. (2011). Atypical necrotizing encephalitis associated with systemic canine distemper virus infection in pups. *Journal of veterinary science*, *12*(4), 409-411.

Millán, J., López-Bao, J. V., García, E. J., Oleaga, Á., Llaneza, L., Palacios, V., De La Torre, A., Rodríguez, A., Dubovi, E. J., & Esperón, F. (2015). Patterns of exposure of Iberian wolves (*Canis lupus*) to canine viruses in human-dominated landscapes. *EcoHealth*, *13*(1), 123-134. https://doi.org/10.1007/s10393-015-1074-8

Mochizuki, M., Hashimoto, M., Hagiwara, S., Yoshida, Y., & Ishiguro, S. (1999). Genotypes of canine distemper virus determined by analysis of the hemagglutinin genes of recent isolates from dogs in Japan. *Journal of Clinical Microbiology*, *37*(9), 2936-2942. https://doi.org/10.1128/JCM.37.9.2936-2942.1999

Molnar, B., Duchamp, C., Moestl, K., Diehl, P.-A., & Betschart, B. (2014). Comparative survey of canine parvovirus, canine distemper virus and canine enteric coronavirus infection in free-ranging wolves of Central Italy and South-Eastern France. *European Journal of Wildlife Research*, *60*(4), 613-624. https://doi.org/10.1007/s10344-014-0825-0

Monne, I., Fusaro, A., Valastro, V., Citterio, C., Pozza, M. D., Obber, F., Trevisiol, K., Cova, M., De Benedictis, P., Bregoli, M., Capua, I., & Cattoli, G. (2011). A distinct CDV genotype causing a major epidemic in Alpine wildlife. *Veterinary Microbiology*, *150*(1-2), 63-69. https://doi.org/10.1016/j.vetmic.2011.01.009

Mourya, D. T., Yadav, P. D., Mohandas, S., Kadiwar, R. F., Vala, M. K., Saxena, A. K., Shete-Aich, A., Gupta, N., Purushothama, P., Sahay, R. R., Gangakhedkar, R. R., Mishra, S. C. K., & Bhargava, B. (2019). Canine distemper virus in Asiatic lions of Gujarat State, India. *Emerging Infectious Diseases*, *25*(11), 2128-2130. https://doi.org/10.3201/eid2511.190120

Müller, A., Silva, E., Santos, N., & Thompson, G. (2011). Domestic dog origin of canine distemper virus in free-ranging wolves in Portugal as revealed by hemagglutinin gene characterization. *Journal of Wildlife Diseases, 47*(3), 725–729. https://doi.org/10.7589/0090-3558-47.3.725

Munkhtsetseg, A., Batmagnai, E., Odonchimeg, M., Ganbat, G., Enkhmandakh, Y., Ariunbold, G., Dolgorsuren, T., Odbileg, R., Dulam, P., Tuvshintulga, B., Sugimoto, C., Sakoda, Y., Yamagishi, J., & Erdenechimeg, D. (2024). Genome sequencing of canine distemper virus isolates from unvaccinated dogs in Mongolia. *The Veterinary Journal, 308*, 106231. https://doi.org/10.1016/j.tvjl.2024.106231

My Van, T., Le, T. Q., & Tran, B. N. (2023). Phylogenetic characterization of the canine distemper virus isolated from veterinary clinics in the Mekong Delta, Vietnam. *Veterinary World*, 1092-1097. https://doi.org/10.14202/vetworld.2023.1092-1097

Namroodi, S., Rostami, A., Majidzadeh-Ardebili, K., Ghalyanchi Langroudi, A., & Morovvati, A. (2015). Detection of Arctic and European cluster of canine distemper virus in north and center of Iran. *Veterinary Research Forum*, *6*(3), 199-204.

Ndiana, L. A., Lanave, G., Vasinioti, V., Desario, C., Martino, C., Colaianni, M. L., Pellegrini, F., Camarda, A., Berjaoui, S., Sgroi, G., Elia, G., Pratelli, A., Buono, F., Martella, V., Buonavoglia, C., & Decaro, N. (2022). Detection and genetic characterization of canine adenoviruses, circoviruses, and novel cycloviruses from wild carnivores in Italy. *Frontiers in Veterinary Science*, *9*, 851987. https://doi.org/10.3389/fvets.2022.851987

Negrão, F. J., Gardinali, N. R., Headley, S. A., Alfieri, A. A., Fernandez, M. A., & Alfieri, A. F. (2013). Phylogenetic analyses of the hemagglutinin gene of wild-type strains of canine distemper virus in southern Brazil. *Genetics and Molecular Research*, *12*(3), 2549-2555. https://doi.org/10.4238/2013.March.11.2

Negrão, F. J., Wosiacki, S. H., Alfieri, A. A., & Alfieri, A. F. (2006). Perfil de restrição de um fragmento do gene da hemaglutinina amplificado pela RT-PCR a partir de estirpes vacinais e selvagens do vírus da cinomose canina. *Arquivo Brasileiro de Medicina Veterinária e Zootecnia*, *58*(6), 1099-1106. https://doi.org/10.1590/S0102-09352006000600019

Nemeth, N. M., Oesterle, P. T., Campbell, G. D., Ojkic, D., & Jardine, C. M. (2018). Comparison of reverse-transcription real-time PCR and immunohistochemistry for the detection of canine distemper virus infection in raccoons in Ontario, Canada. *Journal of Veterinary Diagnostic Investigation*, *30*(2), 319-323. https://doi.org/10.1177/1040638717751825

Nikolin, V. M., Olarte‐Castillo, X. A., Osterrieder, N., Hofer, H., Dubovi, E., Mazzoni, C. J., Brunner, E., Goller, K. V., Fyumagwa, R. D., Moehlman, P. D., Thierer, D., & East, M. L. (2016). Canine distemper virus in the Serengeti ecosystem: molecular adaptation to different carnivore species. *Molecular Ecology, 26*(7), 2111–2130. https://doi.org/10.1111/mec.13902

Nikolin, V. M., Wibbelt, G., Michler, F. F., Wolf, P., & East, M. L. (2011). Susceptibility of carnivore hosts to strains of canine distemper virus from distinct genetic lineages. *Veterinary Microbiology, 156*(1–2), 45–53. https://doi.org/10.1016/j.vetmic.2011.10.009

Oğuzoğlu. (2018). Detection and characterization of distemper virus in a mink (*Neovison vison*) in Turkey. *Veterinaria Italiana*, *54*(1), 79-85. https://doi.org/10.12834/VetIt.936.4787.4

Oleaga, Á., Vázquez, C. B., Royo, L. J., Barral, T. D., Bonnaire, D., Armenteros, J. Á., Rabanal, B., Gortázar, C., & Balseiro, A. (2021). Canine distemper virus in wildlife in South‐Western Europe. *Transboundary and Emerging Diseases*, *69*(4). https://doi.org/10.1111/tbed.14323

Origgi, F. C., Plattet, P., Sattler, U., Robert, N., Casaubon, J., Mavrot, F., Pewsner, M., Wu, N., Giovannini, S., Oevermann, A., Stoffel, M. H., Gaschen, V., Segner, H., & Ryser-Degiorgis, M.-P. (2012). Emergence of canine distemper virus strains with modified molecular signature and enhanced neuronal tropism leading to high mortality in wild carnivores. *Veterinary Pathology*, *49*(6), 913-929. https://doi.org/10.1177/0300985812436743

Özkul, A., Arda Sancak, A., Güngör, E., & Burgu, I. (2004). Determination and phylogenetic analysis of canine distemper virus in dogs with nervous symptoms in Turkey. *Acta Veterinaria Hungarica*, *52*(1), 125-132. https://doi.org/10.1556/avet.52.2004.1.12

Panzera, Y., Calderón, M. G., Sarute, N., Guasco, S., Cardeillac, A., Bonilla, B., Hernández, M., Francia, L., Bedó, G., La Torre, J., & Pérez, R. (2012). Evidence of two co-circulating genetic lineages of canine distemper virus in South America. *Virus Research*, *163*(1), 401-404. https://doi.org/10.1016/j.virusres.2011.10.008

Pardo, I. D. R., Johnson, G. C., & Kleiboeker, S. B. (2005). Phylogenetic characterization of canine distemper viruses detected in naturally infected dogs in North America. *Journal of Clinical Microbiology*, *43*(10), 5009-5017. https://doi.org/10.1128/JCM.43.10.5009-5017.2005

Park, S., Choi, U. S., Kim, E. J., Lee, J. H., Lee, H. B., Cho, H. S., Kim, W., Lim, C. W., & Kim, B. (2016). Coinfection with *Hepatozoon* sp. And Canine Distemper Virus in a Yellow-throated Marten (*Martes flavigula koreana*) in Korea. *Journal of Wildlife Diseases*, *52*(2), 414-417. https://doi.org/10.7589/2015-04-086

Pawar, R. M., Raj, G. D., Gopinath, V. P., Ashok, A., & Raja, A. (2011). Isolation and molecular characterization of canine distemper virus from India. *Tropical Animal Health and Production*, *43*(8), 1617-1622. https://doi.org/10.1007/s11250-011-9880-7

Peserico, A., Marcacci, M., Malatesta, D., Di Domenico, M., Pratelli, A., Mangone, I., D’Alterio, N., Pizzurro, F., Cirone, F., Zaccaria, G., Cammà, C., & Lorusso, A. (2019). Diagnosis and characterization of canine distemper virus through sequencing by MinION nanopore technology. *Scientific Reports*, *9*(1), 1714. https://doi.org/10.1038/s41598-018-37497-4

Pope, J. P., Miller, D. L., Riley, M. C., Anis, E., & Wilkes, R. P. (2016). Characterization of a novel *Canine distemper virus* causing disease in wildlife. *Journal of Veterinary Diagnostic Investigation*, *28*(5), 506-513. https://doi.org/10.1177/1040638716656025

Posuwan, N., Payungporn, S., Thontiravong, A., Kitikoon, P., Amonsin, A., & Poovorawan, Y. (2010). Prevalence of respiratory viruses isolated from dogs in Thailand during 2008-2009. *Asian Biomedicine*, *4*(4), 563-569. https://doi.org/10.2478/abm-2010-0071

Prpić, J., Lojkić, I., Keros, T., Krešić, N., & Jemeršić, L. (2023). Canine distemper virus infection in the free-living wild canines, the red fox (*Vulpes vulpes*) and jackal (*Canis aureus moreoticus*), in Croatia. *Pathogens*, *12*(6). https://doi.org/10.3390/pathogens12060833

Radtanakatikanon, A., Keawcharoen, J., Charoenvisal, N. T., Poovorawan, Y., Prompetchara, E., Yamaguchi, R., & Techangamsuwan, S. (2013). Genotypic lineages and restriction fragment length polymorphism of canine distemper virus isolates in Thailand. *Veterinary Microbiology*, *166*(1-2), 76-83. https://doi.org/10.1016/j.vetmic.2013.05.015

Rahman, D. A., Saepuloh, U., Santosa, Y., Darusman, H. S., Romaria Pinondang, I. M., Kindangen, A. S., Pertiwi, A. P., Sari, L., Irawan, A., Sultan, K., & Rianti, P. (2022). Molecular diagnosis with the corresponding clinical symptoms of canine distemper virus infection in Javan leopard (*Panthera pardus* ssp. *melas*). *Heliyon*, *8*(11), e11341. https://doi.org/10.1016/j.heliyon.2022.e11341

Rätsep, E., & Ojkic, D. (2024). Canine distemper virus infection of vaccinal origin in a 14-week-old puppy. *Journal of Veterinary Diagnostic Investigation*, *36*(2), 287–290. https://doi.org/10.1177/10406387241229436

Rentería-Solís, Z., Förster, C., Aue, A., Wittstatt, U., Wibbelt, G., & König, M. (2014). Canine distemper outbreak in raccoons suggests pathogen interspecies transmission amongst alien and native carnivores in urban areas from Germany. *Veterinary Microbiology*, *174*(1-2), 50-59. https://doi.org/10.1016/j.vetmic.2014.08.034

Ricci, I., Cersini, A., Manna, G., Marcario, G. A., Conti, R., Brocherel, G., Grifoni, G., Eleni, C., & Scicluna, M. T. (2021). A canine distemper virus retrospective study conducted from 2011 to 2019 in Central Italy (Latium and Tuscany regions). *Viruses*, *13*(2), 272. https://doi.org/10.3390/v13020272

Richards, S. M., Rainwater, K. A. E., Stephens, J. R., & Rainwater, T. R. (2008). An observation of aberrant behavior in a raccoon (*Procyon lotor*) infected with canine distemper virus. *Southeastern Naturalist*, *7*(3), 556-558. https://doi.org/10.1656/1528-7092-7.3.556

Riley, M. C., & Wilkes, R. P. (2015). Sequencing of emerging canine distemper virus strain reveals new distinct genetic lineage in the United States associated with disease in wildlife and domestic canine populations. *Virology Journal*, *12*(1), 219. https://doi.org/10.1186/s12985-015-0445-7

Rodríguez-Cabo-Mercado, R., Martínez-Hernández, F., Aréchiga-Ceballos, N., López-Diaz, O., Irais Muñoz-García, C., Aguilar-Setien, A., Villalobos, G., Villanueva-García, C., Verdugo-Rodríguez, A., Iturbe-Ramírez, R., & Rendon-Franco, E. (2020). Canine distemper in neotropical procyonids: molecular evidence, humoral immune response and epidemiology. *VIRUS RESEARCH*, *290*, 198164. https://doi.org/10.1016/j.virusres.2020.198164

Romanutti, C., Gallo Calderon, M., Keller, L., Mattion, N., & La Torre, J. (2016). RT-PCR and sequence analysis of the full-length fusion protein of canine distemper virus from domestic dogs. *Journal of Virological Methods*, *228*, 79-83. https://doi.org/10.1016/j.jviromet.2015.11.011

Rosa, G. M., Santos, N., Grøndahl-Rosado, R., Fonseca, F. P., Tavares, L., Neto, I., Cartaxeiro, C., & Duarte, A. (2020). Unveiling patterns of viral pathogen infection in free-ranging carnivores of northern Portugal using a complementary methodological approach. *Comparative Immunology, Microbiology and Infectious Diseases*, *69*, 101432. https://doi.org/10.1016/j.cimid.2020.101432

Rosa, G. N., Domingues, H. G., Felippe, P. A. N., & Spilki, F. R. (2012). Detecção molecular e análise ϐilogenética do gene H de amostras do vírus da cinomose canina em circulação no município de Campinas, São Paulo. *Pesquisa Veterinaria Brasileira*, *32*(1), 72-77.

Rzezutka, A., & Mizak, B. (2002). Application of N-PCR for diagnosis of distemper in dogs and fur animals. *Veterinary Microbiology*, *88*(1), 95-103. https://doi.org/10.1016/s0378-1135(02)00097-4

Sarute, N., Pérez, R., Aldaz, J., Alfieri, A. A., Alfieri, A. F., Name, D., Llanes, J., Hernández, M., Francia, L., & Panzera, Y. (2014). Molecular typing of canine distemper virus strains reveals the presence of a new genetic variant in South America. *Virus Genes*, *48*(3), 474-478. https://doi.org/10.1007/s11262-014-1054-z

Schatzberg, S. J., Li, Q., Porter, B. F., Barber, R. M., Claiborne, M. K., Levine, J. M., Levine, G. J., Israel, S. K., Young, B. D., Kiupel, M., Greene, C., Ruone, S., Anderson, L., & Tong, S. (2009). Broadly reactive pan-paramyxovirus reverse transcription polymerase chain reaction and sequence analysis for the detection of *canine distemper virus* in a case of canine meningoencephalitis of unknown etiology. *Journal of Veterinary Diagnostic Investigation*, *21*(6), 844-849. https://doi.org/10.1177/104063870902100613

Seimon, T. A., Miquelle, D. G., Chang, T. Y., Newton, A. L., Korotkova, I., Ivanchuk, G., Lyubchenko, E., Tupikov, A., Slabe, E., & McAloose, D. (2013). Canine distemper virus: an emerging disease in wild endangered Amur tigers (*Panthera tigris altaica*). *mBio*, *4*(4). https://doi.org/10.1128/mBio.00410-13

Sekulin, K., Hafner-Marx, A., Kolodziejek, J., Janik, D., Schmidt, P., & Nowotny, N. (2011). Emergence of canine distemper in Bavarian wildlife associated with a specific amino acid exchange in the haemagglutinin protein. *The Veterinary Journal*, *187*(3), 399-401. https://doi.org/10.1016/j.tvjl.2009.12.029

Shi, N., Zhang, L., Yu, X., Zhu, X., Zhang, S., Zhang, D., & Duan, M. (2021). insight into an outbreak of canine distemper virus infection in masked palm civets in China. *Frontiers in Veterinary Science*, *8*, 728238. https://doi.org/10.3389/fvets.2021.728238

Shin, Y.-S., Mori, T., Okita, M., Gemma, T., Kai, C., & Mikami, T. (1995). Detection of canine distemper virus nucleocapsid protein gene in canine peripheral blood mononuclear cells by RT-PCR. *Journal of Veterinary Medical Science*, *57*(3), 439-445.

Simon-Martinez, J., Ulloa-Arvizu, R., Soriano, V. E., & Fajardo, R. (2008). Identification of a genetic variant of canine distemper virus from clinical cases in two vaccinated dogs in Mexico. *Veterinary Journal*, *175*(3), 423-426. https://doi.org/10.1016/j.tvjl.2007.01.015

Șonea, C., Gurău, M. R., Sersea, M. S., Crețu, D. M., Ștefan, G., Oțelea, F., & Bărăităreanu, S. (2023). Nucleic acid amplification testing of urine samples from dogs with distemper-like disease. *Revista Romana de Medicina Veterinara*, *33* (1), 69-74.

Stanton, J. B., Brown, C. C., Poet, S., Lipscomb, T. P., Saliki, J., & Frasca, S. (2004). Retrospective differentiation of canine distemper virus and phocine distemper virus in phocids. *Journal of Wildlife Diseases*, *40*(1), 53-59. https://doi.org/10.7589/0090-3558-40.1.53

Stilwell, J. M., Anis, E., Wilkes, R. P., & Rissi, D. R. (2019). Dual infection with an emergent strain of canine distemper virus and canine parvovirus in an Arctic wolf under managed care. *Journal of Veterinary Diagnostic Investigation*, *31*(4), 594-597. https://doi.org/10.1177/1040638719851832

Stimmelmayr, R., Rotstein, D. S., Maboni, G., Person, B. T., & Sanchez, S. (2018). Morbillivirus-associated lipid pneumonia in Arctic foxes. *Journal of Veterinary Diagnostic Investigation*, *30*(6), 933-936. https://doi.org/10.1177/1040638718797382

Sun, Z., Li, A., Ye, H., Shi, Y., Hu, Z., & Zeng, L. (2010). Natural infection with canine distemper virus in hand-feeding Rhesus monkeys in China. *Veterinary Microbiology*, *141*(3-4), 374-378. https://doi.org/10.1016/j.vetmic.2009.09.024

Tan, B., Wen, Y., Wang, F., Zhang, S., Wang, X., Hu, J., Shi, X., Yang, B., Chen, L., Cheng, S., & Wu, H. (2011). Pathogenesis and phylogenetic analyses of canine distemper virus strain ZJ7 isolate from domestic dogs in China. *Virology Journal, 8*(1). https://doi.org/10.1186/1743-422x-8-520

Thomas, N., White, C. L., Saliki, J., Schuler, K., Lynch, D., Nielsen, O., Dubey, J. P., & Knowles, S. (2020). Canine distemper virus in the sea otter (*Enhydra lutris*) population in Washington state, USA. *Journal of Wildlife Diseases*, *56*(4). https://doi.org/10.7589/JWD-D-19-00008

Timm, S. F., Munson, L., Summers, B. A., Terio, K. A., Dubovi, E. J., Rupprecht, C. E., Kapil, S., & Garcelon, D. K. (2009). A suspected canine distemper epidemic as the cause of a catastrophic decline in Santa Catalina Island foxes (*Urocyon littoralis catalinae*). *Journal of Wildlife Diseases*, *45*(2), 333-343. https://doi.org/10.7589/0090-3558-45.2.333

Tomaszewicz Brown, A. T., McAloose, D., Calle, P. P., Auer, A., Posautz, A., Slavinski, S., Brennan, R., Walzer, C., & Seimon, T. A. (2020). Development and validation of a portable, point-of-care canine distemper virus qPCR test. *PLoS One*, *15*(4), e0232044. https://doi.org/10.1371/journal.pone.0232044

Trogu, T., Canziani, S., Salvato, S., Bianchi, A., Bertoletti, I., Gibelli, L. R., Alborali, G. L., Barbieri, I., Gaffuri, A., Sala, G., Sozzi, E., Lelli, D., Lavazza, A., & Moreno, A. (2021). Canine distemper outbreaks in wild carnivores in Northern Italy. *Viruses*, *13*(1), 99. https://doi.org/10.3390/v13010099

Van De Bildt, M. W. G. (2002). Distemper outbreak and its effect on African wild dog conservation. *Emerging Infectious Diseases*, *8*(2), 212-213. https://doi.org/10.3201/eid0802.010314

Verna, F., Giorda, F., Miceli, I., Rizzo, G., Pautasso, A., Romano, A., Iulini, B., Pintore, M. D., Mignone, W., Grattarola, C., Bozzetta, E., Varello, K., Dondo, A., Casalone, C., & Goria, M. (2017). Detection of morbillivirus infection by RT-PCR RFLP analysis in cetaceans and carnivores. *Journal of Virological Methods*, *247*, 22-27. https://doi.org/10.1016/j.jviromet.2017.05.009

Wang, F., Yan, X., Chai, X., Zhang, H., Zhao, J., Wen, Y., & Wu, W. (2011). Differentiation of canine distemper virus isolates in fur animals from various vaccine strains by reverse transcription-polymerase chain reaction-restriction fragment length polymorphism according to phylogenetic relations in China. *Virology Journal*, *8*(1). https://doi.org/10.1186/1743-422x-8-85

Wang, J., Luo, Y., Liang, L., Li, J., & Cui, S. (2018). A fast and simple one-step duplex PCR assay for canine distemper virus (CDV) and canine coronavirus (CCoV) detection. *Archives of Virology*, *163*(12), 3345-3349. https://doi.org/10.1007/s00705-018-3982-8

Wang, J., Wang, J., Li, R., Shi, R., Liu, L., & Yuan, W. (2018). Evaluation of an incubation instrument-free reverse transcription recombinase polymerase amplification assay for rapid and point-of-need detection of canine distemper virus. *Journal of Virological Methods*, *260*, 56-61. https://doi.org/10.1016/j.jviromet.2018.07.007

Wang, R., Wang, X., Zhai, J., Zhang, P., Irwin, D. M., Shen, X., Chen, W., & Shen, Y. (2022). A new canine distemper virus lineage identified from red pandas in China. *Transboundary and Emerging Diseases,* *69*(4). https://doi.org/10.1111/tbed.14370

Wang, L.-C., Kuo, Y.-T., Chueh, L.-L., Huang, D., & Lin, J.-H. (2017). The detection and differentiation of canine respiratory pathogens using oligonucleotide microarrays. *Journal of Virological Methods*, *243*, 131-137. https://doi.org/10.1016/j.jviromet.2017.02.004

Weber, M. N., Mosena, A. C. S., da Silva, M. S., Canova, R., de Lorenzo, C., Olegario, J. C., Budaszewski, R. F., Baumbach, L. F., Soares, J. F., Sonne, L., Varela, A. P. M., Mayer, F. Q., de Oliveira, G. S., & Canal, C. W. (2020). Virome of crab-eating (*Cerdocyon thous*) and pampas foxes (*Lycalopex gymnocercus*) from southern Brazil and Uruguay. *Infection Genetics and Evolution*, *85*, 104421. https://doi.org/10.1016/j.meegid.2020.104421

Weckworth, J. K., Davis, B. W., Dubovi, E., Fountain‐Jones, N., Packer, C., Cleaveland, S., Craft, M. E., Eblate, E., Schwartz, M., Mills, L. S., & Roelke‐Parker, M. (2020). Cross‐species transmission and evolutionary dynamics of canine distemper virus during a spillover in African lions of Serengeti National Park. *Molecular Ecology, 29*(22), 4308–4321. https://doi.org/10.1111/mec.15449

Wilkes, R. P., Sanchez, E., Riley, M. C., & Kennedy, M. A. (2014). Real-time reverse transcription polymerase chain reaction method for detection of *Canine distemper virus* modified live vaccine shedding for differentiation from infection with wild-type strains. *Journal of Veterinary Diagnostic Investigation*, *26*(1), 27-34. https://doi.org/10.1177/1040638713517232

Willi, B., Spiri, A. M., Meli, M. L., Grimm, F., Beatrice, L., Riond, B., Bley, T., Jordi, R., Dennler, M., & Hofmann-Lehmann, R. (2015). Clinical and molecular investigation of a canine distemper outbreak and vector-borne infections in a group of rescue dogs imported from Hungary to Switzerland. *BMC Veterinary Research*, *11*, 154. https://doi.org/10.1186/s12917-015-0471-0

Woma, T. Y., Van Vuuren, M., Bosman, A.-M., Quan, M., & Oosthuizen, M. (2010). Phylogenetic analysis of the haemagglutinin gene of current wild-type canine distemper viruses from South Africa: lineage Africa. *Veterinary Microbiology*, *143*(2-4), 126-132. https://doi.org/10.1016/j.vetmic.2009.11.013

Wostenberg, D. J., Walker, N., Fox, K. A., Spraker, T. R., Piaggio, A. J., & Gilbert, A. (2018). Evidence of two cocirculating canine distemper virus strains in mesocarnivores from Northern Colorado, USA *Journal of Wildlife Diseases*, *54*(3), 534-543. https://doi.org/10.7589/2017-09-238

Wu, L., Sun, Y., Xu, C., Li, C., & Xia, C. (2015). Molecular cloning and sequence analysis of hemagglutinin gene of a novel strain canine distemper virus. *International Journal of Applied Research in Veterinary Medicine*, *13*(1), 36-41.

Yi, L., Cheng, S., Xu, H., Wang, J., Cheng, Y., Yang, S., & Luo, B. (2012). Development of a combined canine distemper virus specific RT-PCR protocol for the differentiation of infected and vaccinated animals (DIVA) and genetic characterization of the hemagglutinin gene of seven Chinese strains demonstrated in dogs. *Journal of Virological Methods*, *179*(1), 281-287. https://doi.org/10.1016/j.jviromet.2011.11.011

Yılmaz, V., Coşkun, N., Timurkan, M. Ö., Karakurt, E., Nuhoğlu, H., Erkılıç, E. E., Kırmızıgül, A. H., & Sezer, M. (2022). The Investigation of canine distemper virus in different diagnosis materials of dogs using molecular and pathological methods, Northeastern Turkey. *Indian Journal of Animal Research*, *Of*. https://doi.org/10.18805/IJAR.B-1389

Young, K. T., Lahmers, K. K., Sellers, H. S., Stallknecht, D. E., Poulson, R. L., Saliki, J. T., Tompkins, S. M., Padykula, I., Siepker, C., Howerth, E. W., Todd, M., & Stanton, J. B. (2021). Randomly primed, strand-switching MinION-based sequencing for the detection and characterization of cultured RNA viruses. *Journal of Veterinary Diagnostic Investigation, 33*(2), 202-215. https://doi.org/10.1177/1040638720981019

Zhang, H., Meng, P., Song, X., Li, S., Yang, R., Zhang, C., Shan, H., & Wen, Y. (2021). Isolation and phylogenetic analysis of the canine distemper virus from a naturally infected dog in China. *Indian Journal of Animal Research*. https://doi.org/10.18805/IJAR.B-1298

Zhao, J., Yan, X., Chai, X., Martella, V., Luo, G., Zhang, H., Gao, H., Liu, Y., Bai, X., Zhang, L., Chen, T., Xu, L., Zhao, C., Wang, F., Shao, X., Wu, W., & Cheng, S. (2010). Phylogenetic analysis of the haemagglutinin gene of canine distemper virus strains detected from breeding foxes, raccoon dogs and minks in China. *Veterinary Microbiology*, *140*(1–2), 34–42. https://doi.org/10.1016/j.vetmic.2009.07.010
