# Supplementary material for: Geographical Distribution of Carnivore Hosts and Genotypes of Canine Distemper Virus (CDV) Worldwide: A Scoping Review and Spatial Meta-Analysis
Source: Transbound Emerg Dis. 2025 Mar 5;2025:6632068. doi: 10.1155/tbed/6632068 (PMC12016734; doi:10.1155/tbed/6632068)
Supplement: Supporting Information 3 — A table with extra information about Morbillivirus canis (CDV) genotypes used in this scoping review. Other lineages/genotypes included in every category are specified: number of records in which every genotype appears; percentage of occurrence of every genotype out of the total of 303 records that classified the CDV genetic material into one of the main genotypes covered; the period of years in which every genotype was published; study citing every genotype for the first time; the oldest detected strain of every genotype: year of detection, species, country, study; most recent study citing every genotype; the most recent detected strain of every genotype: year of detection, species, country, study. [file 6632068.f3.docx]

**Supplementary File 3.** Categories of Canis distemper virus (CDV) genotypes included in this scoping review. A total of 160 articles published between 1985 and 2024 were analyzed. A total of 457 records were categorized by positive animal species, sequenced genotype, and location where the published genotype was detected. 398 records detected CDV by PCR. Of these, 332 records sequenced the detected genetic material; of these, 303 records were able to classify CDV into one of the main classified genotypes.

| **Genotype** | **Other lineages included^1^** | **N^2^** | **%^3^** | **Year^4^** | **First study^5^** | **First strain sequenced^6^** | **Last study^7^** | **Last strain sequenced^8^** |
| --- | --- | --- | --- | --- | --- | --- | --- | --- |
| **America-1** | Western vaccine, Rockborn like, North America-1 | 34 | 11.22 | 1999-2024 | Frisk et al., 1999^9^ | 1999, dog (*Canis lupus familiaris*), Germany | Rätsep & Ojkic, 2024^10^ | 2024, dog, Canada |
| **America-2** |  | 9 | 2.97 | 2007-2019 | Demeter et al., 2007 [67] | 1989, Canadian lynx (*Lynx canadensis*) and bobcat (*Lynx rufus*), Canada (Daoust et al., 2009) [70] | Young et al., 2021 [135] | 2018, racoon (*Procyon lotor*), USA |
| **America-3** | North America-3 | 3 | 0.99 | 2014-2018 | Sarute et al., 2014 [71] | 2006, dog, Ecuador | Anis et al., 2018 [72] | 2014-2017, dog, USA |
| **America-4** | South America/North America-4 | 10 | 3.30 | 2015-2022 | Riley & Wilkes, 2015 [73] | 2010, dog, red fox (*Vulpes vulpes*) and racoon, USA | Echeverry-Bonilla et al., 2022 [34] | South America/North America-4 (3 records): Duque Valencia et al., 2019 [74], strain from 2017, Colombia; Echeverry-Bonilla et al., 2022 [34], strain from 2021, Crab-eating fox (*Cerdocyon thous*), Colombia. America-4 (4 records): Pope et al., 2016 [18], strain from 2013, gray fox (*Urocyon cinereoargenteus*), USA. North America-4 (3 records): Riley & Wilkes, 2015 [73], strains from 2010, dog, red fox and racoon, USA. |
| **Europe/South America-1** | Genotypes reported as Europe-1 (52 records): from 2001 (Martella et al., 2002) [56] to 2024 (Huang et al., 2024) [57] Genotypes reported as Europe/South America-1 (31 records): from 2009 (Megid et al., 2009) [58] to 2023 (Ndiana et al., 2023) [60] | 83 | 27.39 | 2001-2024 | Martella et al., 2002 [56] | Europe-1: 2001, red fox (*Vulpes vulpes*), Italy, Martella et al., 2002 [56].  Europe/South America-1: 1997, Steppe polecat *(Mustela eversmanii*), European polecat (*Mustela putorius*), beech marten (*Martes foina*), pine marten (*Martes martes*), least weasel (*Mustela nivalis*) and stoat (*Mustela erminea*), Hungary, Lanzski et al., 2022. [153]. In South America: 2006, dog, Brazil and Uruguay and Ecuador Sarute et al.,2014 [31] | Europe-1: Huang et al., 2024 [57]; Europe/South America-1: Ndiana et al., 2023 [60] | Europe-1: 2024, badger *(Meles meles),* Huang et al., 2024 [57].  Europe/South America-1: 2023, dog, Nigeria, Ndiana et al., 2023 [60]. |
| **South America-2** | South America 2-Argentina. Other strains from Argentina | 5 | 1.65 | 2007-2020 | Gallo Calderón et al., 2007 [75] | 2003, dog, Argentina | Kodi et al., 2020 [78] | 2019, dog, India |
| **South America-3** | Other Colombia strains | 5 | 1.65 | 2014-2019 | Espinal et al., 2014 [59] | 2011, dog, Colombia | Duque Valencia et al., 2019 [79] | 2017, dog, Colombia |
| **Europe-2/European Wildlife** | Europe-2 | 23 | 7.59 | 2008-2022 | Kapil et al., 2008 [66] | 2006, beech marten, badger and red fox, Italy, Monne et al., 2011 [41] | Di Francesco et al., 2022 [130] | 2021, brown bear (*Ursus arctos marsicanus*), dog and red fox, Italy |
| **Europe-3/Arctic** | Arctic-like | 47 | 15.51 | 2003-2024 | Maes et al., 2003 [62] | 2000, dog, USA, Martella et al., 2006 [36] | Glišić et al., 2024 [121] | 2019, golden jackal (*Canis aureus*), Serbia. |
| **Asia-1** |  | 44 | 14.52 | 2007-2024 | Martella et al., 2007 [142] | 1997, beech marten, South Korea, An et al., 2008 [82] | Munkhtsetseg et al., 2024 [88] | 2023, dog, Vietnam, Van et al., 2023 [85] |
| **Asia-2** |  | 3 | 0.99 | 2005-2008 | Lan et al., 2005 [85] | Dog, Japan | An et al., 2008 [82] | 1997-1998, dog, South Korea |
| **Asia-3** |  | 5 | 1.65 | 2010-2020 | Zhao et al., 2010 [64] | 2010, red fox, China | Kadam et al., 2020 [45] | 2020, Asiatic lion (*Panthera leo persica*), palm civet (*Paradoxurus hermaphroditus*), India |
| **Asia-4** |  | 4 | 1.32 | 2013-2024 | Radtanakatikanon et al., 2013 [84] | 2010, dog, Thailand | Munkhtsetseg et al., 2024 [88] | 2021 dog, Mongolia. From 2013 (Radtanakatikanon et al., 2013 [84], 2010 dog, Thailand), 2015 (Bi et al., 2015 [90], 2011 strain, China) till 2020 (Kodi et al., 2021 [78], 2019 dog, India) |
| **Asia-5** |  | 3 | 0.99 | 2019-2020 | Bhatt et al., 2019 [35] | 2015, dog, India | Manandhar et al., 2023 [91] | 2018, dog, Nepal |
| **Asia-6** |  | 2 | 0.66 | 2005-2022 | Pardo et al., 2005 [92] | 2004, dog, USA | Wang et al., 2022 [139] | 2018, Lesser panda (*Ailuropoda fulgens*), China. |
| **Africa-1** | Africa-1/Southern Africa genotypes | 10 | 3.30 | 2009-2019 | Gowtage et al., 2009 [94] | 2001, dog, black-backed jackal (*Lupulella mesomelas*), Namibia | Mourya et al., 2019 [95] | 2018, Asiatic lion, India |
| **Africa-2** | Africa-2/Eastern Africa genotypes | 13 | 4.29 | 1995-2020 | Harder et al., 1995 [96]; Weckworth et al.,2020 [97] | 1993-1994, African lion (*Panthera leo*), spotted hyena (*Crocuta crocuta*), dog, bat-eared fox (*Otocyon megalotis*), Tanzania | Nikolin et al., 2017 [30] | 2007, African wild dog (*Lycaon pictus*), Tanzania; 2011, golden jackal, Tanzania. |
| **Total** | | 303 |  |  |  |  |  |  |

(1) Other lineages/genotypes included in this category are specified. (2) Number of records in which this genotype appears. (3) Percentage of occurrence of the genotype out of the total of 303 records that classified the CDV genetic material into one of the main genotypes covered. (4) Time span of years in which this genotype is published. (5) Study citing this genotype for the first time. (6) Oldest detected strain of this genotype: year of detection, species, country, study (may or may not coincide with the study citing for the first time). (7) Most recent study citing this genotype. (8) Most recent detected strain of this genotype: year of detection, species, country, study (may or may not coincide with the study last cited). (9) Frisk, A. L., König, M., Moritz, A., & Baumgärtner, W. (1999). Detection of canine distemper virus nucleoprotein RNA by reverse transcription-PCR using serum, whole blood, and cerebrospinal fluid from dogs with distemper. *Journal of clinical microbiology*, *37*(11), 3634–3643. <https://doi.org/10.1128/JCM.37.11.3634-3643.1999>. (10) Rätsep, E., & Ojkic, D. (2024). Canine distemper virus infection of vaccinal origin in a 14-week-old puppy. *Journal of veterinary diagnostic investigation: official publication of the American Association of Veterinary Laboratory Diagnosticians, Inc*, *36*(2), 287–290. <https://doi.org/10.1177/10406387241229436>.
